# Doxorubicin—An Agent with Multiple Mechanisms of Anticancer Activity

**DOI:** 10.3390/cells12040659

**Published:** 2023-02-19

**Authors:** Mateusz Kciuk, Adrianna Gielecińska, Somdutt Mujwar, Damian Kołat, Żaneta Kałuzińska-Kołat, Ismail Celik, Renata Kontek

**Affiliations:** 1 Department of Molecular Biotechnology and Genetics, University of Lodz, 90-237 Lodz, Poland; 2 Doctoral School of Exact and Natural Sciences, University of Lodz, 90-237 Lodz, Poland; 3 Chitkara College of Pharmacy, Chitkara University, Rajpura 140401, Punjab, India; 4 Department of Experimental Surgery, Faculty of Medicine, Medical University of Lodz, 90-136 Lodz, Poland; 5 Department of Pharmaceutical Chemistry, Faculty of Pharmacy, Erciyes University, 38039 Kayseri, Turkey

**Keywords:** adriamycin, apoptosis, DNA damage response (DDR), doxorubicin, immunotherapy

## Abstract

Doxorubicin (DOX) constitutes the major constituent of anti-cancer treatment regimens currently in clinical use. However, the precise mechanisms of DOX’s action are not fully understood. Emerging evidence points to the pleiotropic anticancer activity of DOX, including its contribution to DNA damage, reactive oxygen species (ROS) production, apoptosis, senescence, autophagy, ferroptosis, and pyroptosis induction, as well as its immunomodulatory role. This review aims to collect information on the anticancer mechanisms of DOX as well as its influence on anti-tumor immune response, providing a rationale behind the importance of DOX in modern cancer therapy.

## 1. Introduction

Drugs used in chemotherapy can be categorized into several groups according to a variety of criteria, including their function and chemical framework. Depending on the mode of action, they can be categorized as alkylating agents, antimetabolites, topoisomerase inhibitors, mitotic spindle inhibitors, and others [1,2,3]. Topoisomerase I inhibitors, such as irinotecan and topotecan, and topoisomerase II inhibitors, such as etoposide, teniposide, and anthracyclines (idarubicin, daunorubicin, and doxorubicin (DOX)), induce DNA strand breaks and hinder the action of topoisomerases that are involved in the DNA replication and process of transcription [3]. DOX has been demonstrated to have significant therapeutic potential and is recognized as one of the most efficient chemotherapy medications that have been approved by the Food and Drug Administration (FDA) for the treatment of various cancers such as breast cancer, carcinomas, sarcomas, and hematological malignancies [4]. Despite the extensive application of anthracyclines, the toxic effects of these drugs are multifaceted, with cardiotoxicity being the most well-known and most comprehensively studied adverse effect, as reviewed by multiple authors [4,5,6,7,8,9,10]. Moreover, damage to other organs [11,12,13], including the brain [12,14,15], liver [16], and kidneys [17,18,19], can also occur. It is already known that the anticancer activity of DOX can be attributed to the capability of the drug to intercalate into DNA, inhibit topoisomerase II, disrupt mitochondria function, and potentiate free-radical generation and oxidative damage. However, the precise mechanisms of action of DOX are complex and still relatively unknown [4,12,20]. There have been many initiatives attempted to lessen the adverse effects of DOX, including the use of substances that possess antioxidant and/or antiapoptotic activity [17,21,22,23,24,25], the creation of efficient delivery systems [12,26,27,28,29,30,31,32], prodrugs [33,34,35,36,37,38], and the development of DOX analogues [39,40,41,42,43], as recently reviewed by Sohali et al. [44]. On the other hand, some of these techniques were not successful in reducing the toxic effects of anthracycline when tested in animal models or in human clinical trials [45,46,47,48,49,50,51]. It is important to make an effort to find more efficient methods of combating DOX toxicity while maintaining or strengthening its therapeutic effects [4].

Despite the side effects induced by DOX it is still widely used in cancer therapy, predominantly in new formulations (mentioned above) or drug combinations [52,53,54,55,56]. In addition to their cytotoxic effect, chemotherapeutic drugs may also boost the infiltration of CD8^+^ T cells and neutral killer (NK) cells into tumors, as well as the maturation of antigen-presenting cells (APCs), such as tumor macrophages or dendritic cells. Primary cytostatic and cytotoxic medicines function in this way to restore an immune-reactive tumor microenvironment, which ultimately increases the tumor’s susceptibility to immunotherapy. Moreover, many of the medicines that are presently in use have not been investigated in terms of their potential to activate the immune system against tumor cells or for their value in combinations with immune checkpoint inhibitors [57].

Here we review new insights into the DOX mode of action including its contribution to DNA damage, reactive oxygen species (ROS) production, apoptosis, senescence, autophagy, ferroptosis, and pyroptosis induction, as well as the emerging influence of this anthracycline drug on the immune system and antitumor immune response.

## 2. Mode of Action

### 2.1. Induction of DNA Damage

#### 2.1.1. Formation of DOX–DNA Adducts and DNA Intercalation

Like other anthracyclines, DOX intercalates DNA through the formation hydrogen bonds with guanines in adjacent GC base pairs [58,59]; however, the exact mechanism of this interaction is not completely understood. One of the postulated mechanisms to explain the effect of DOX on malignant cells is that the intercalation of DOX into DNA untwists the molecule and results in positive supercoiling of the DNA helix [60,61]. There is also evidence that DOX increases the turnover of nucleosomes surrounding promoters of active genes due to its intercalation and induces changes in DNA topology [61,62]. It is possible that the unwinding of DNA that occurs as a result of DOX intercalation provides a significant amount of positive torsional stress that destabilize nucleosomes [63].

Moreover, it has been demonstrated that the formation of DOX–DNA adducts can activate the DNA damage response (DDR) pathway independently of the effect of the drug on topoisomerase II. Importantly, DOX–DNA adducts can be detected at drug doses that are appropriate to clinical practice, which suggests that DOX–DNA adducts can be formed when chemotherapy is applied [64,65,66]. However, the formation of adducts seem not to be the primary action mechanism of DOX [66].

#### 2.1.2. Topoisomerase Trapping

During DNA replication and transcription, topoisomerases play a crucial role in the preservation of the correct DNA structure. Supercoiled DNA emerging during the above-mentioned processes can unwind and efficiently function as a template upon the introduction of single- or double-strand breaks (SSBs or DSBs) by topoisomerase type I and II enzymes, respectively [67]. Topoisomerase II enzyme is possibly the main target of DOX; however, topoisomerase I inhibition may also play a role in DOX cytotoxicity. Low dosages (<1 µM) of DOX are believed to capture covalently bound topoisomerase II at DNA ds-breaking sites and inhibit DNA relegation [68,69]. Furthermore, the treatment of cancer cells with DOX induces up-regulation of genes of the DDR pathway [61] as a consequence of the induction of SSBs and DSBs in the DNA molecule [70].

Ataxia telangiectasia and Rad3-related (ATR) was discovered in 1996 as the mammalian orthologue of mitotic entry checkpoint protein 1, also known as Mec1. Mec1 is the primary kinase that is responsible for the coordination of DNA damage checkpoints in budding yeast. Structurally, ATR is a phosphoinositide 3-kinase-related protein kinase (PIKK) essential for cell viability due to its major role in the coordination of pathways involved in the replication stress response [71,72,73]. ATR can react to a wide variety of genotoxic stresses, such as those caused by UV radiation, DNA polymerase inhibitors, or topoisomerase poisons. One characteristic that is shared by all of these stressors is the fact that they stop or delay DNA polymerases in replication forks [74]. ATR detects single-stranded DNA (ssDNA) emerging at halted replication forks as a result of minichromosome maintenance (MCM2-7)-complex helicase activity. ATR kinase present in cells as an inactive dimer becomes active upon autophosphorylation reaction in response to DNA damage emergence. Replication protein A/ATR-interacting protein (RPA-ATRIP) complexes are generated on single-stranded DNA and act as a platform for the ATR kinase. Moreover, topoisomerase 2-binding protein 1 (TOPBP1) and the RAD9-RAD1-HUS1 components of the 9-1-1 cell-cycle checkpoint control protein complex are also necessary for the complete activation of the kinase [75,76,77,78,79]. Furthermore, additional factors including the MRE11–RAD50–NBS1 (MRN) complex and RAD9, RAD1, HUS1 interacting nuclear orphan (RHINO) may be involved in the recruitment of TOPBP1 for efficient activation of the ATR kinase [74]. Recent research has shown that Ewing’s tumor-associated antigen 1 (ETAA1) protein also has an ATR-activating domain. In contrast to the embryonically lethal effects of a TOPBP1 impairment in mice, the carriers of ETAA1 mutations are viable [71,72].

SSBs are detected by the poly (ADP-ribose) polymerase 1 (PARP1), which associates with the ends of the strand breaks and creates complexes with tyrosyl-DNA phosphodiesterase 1 (TDP-1), X-ray repair cross-complementing group-1 (XRCC1), and polynucleotide kinase phosphatase (PNKP). The above-mentioned factors are recruited to the sites of SSB for efficient removal of non-canonical DNA ends. For example, TDP1 catalyzes the conversion of 3′-phosphotyrosyl bonds to the 3′-OH ends, while PNKP removes the phosphate groups from the 3′-end and catalyzes the phosphorylation of the 5′-hydroxyl groups of DNA, reconstructing the regular (3′-OH and 5′-P) DNA strand ends [78,80]. Nonetheless, PARP-1 constitutes the central hub protein of the response to single-strand DNA breaks and single-strand break repair (SSBR). PARP-1 is involved in the recognition and signaling of occurring damage and catalyzes an evolutionarily conserved enzymatic reaction that involves attachment of poly (ADP-ribose) polymers (PARs) to arginine, glutamate, aspartate, cysteine, lysine, and serine residues of many adaptor proteins and histones at the sites of DNA damage. This leads to the efficient recruitment of DNA repair factors to the relaxed chromatin [76,78,81,82,83,84], including cellular tumor antigen p53 (TP53), DNA ligase III, XRCC1, DNA-dependent protein kinase catalytic subunit (DNA-PK_CS_), and KU70/80 [84]. 

Upon detection of DSBs, cells respond by rapidly and precisely relocating a wide variety of proteins to the area around the lesion. These proteins assemble into massive, overlapping multi-subunit structures called foci. Sequestration of proteins in the DSB’s immediate vicinity may play a role in increasing the damage-induced checkpoint signal and easing the repair of persistent damage [85]. DSBs are detected and processed by the MRN complex. The meiotic recombination 11 homolog 1 (MRE11) complex component works as both a DNA endonuclease and a 3′–5′ dsDNA exonuclease that mediates the resection of aberrant DNA structures such as hairpins, while RAD50 and nibrin (NBS1) stimulate the activity of the nuclease. The MRN concentration at the site of DNA damage serves as a signal for ataxia telangiectasia mutated A-T mutated (ATM) recruitment [85,86,87]. ATM, similar to ATR, exists in cells as an inactive dimer that undergoes monomerization in response to DNA damage. Activated ATM phosphorylates exposed H2AX histone proteins in the vicinity of DSBs, leading to the accumulation of phosphorylation on Ser-139 H2AX (γ-H2AX) histone foci, which serve as an anchorage for the platform protein mediator of DNA damage checkpoint protein 1 (MDC-1). The recruitment of MDC-1 contributes to the further accumulation of ATM at the damage site and amplification of the signal through phosphorylation of H2AX proteins and accumulation of MDC-1 [76,78].

DNA-PKcs constitutes another DDR central hub protein along with ATR and ATM kinases and also belongs to the PIKK family. In contrast to the ATM-mediated pathway, which facilitates DNA repair through the homologous recombination pathway (HR), the detection of DSBs by KU70/80 proteins and formation of the repair complexes with DNA-PKcs favors the repair of occurring breaks by the non-homologous end joining (NHEJ) pathway. Besides its role in the canonical NHEJ pathway, DNA-PKcs participates in replication stress through the phosphorylation of ATR-activating RPA32 protein on Ser-4 and -8 and subsequent activation of the replication checkpoints [88,89] described below.

In the following events, ATR and ATM phosphorylate checkpoint kinases (CHK1 and CHK2), which mediate the downstream events of the DDR. Checkpoint kinases phosphorylate M-phase inducer phosphatases (CDC25A and CDC25C), which control cell-cycle progression through the dephosphorylation of cyclin–cyclin-dependent kinase (CDK) complexes. CDK4 and CDK6 become active following exposure to mitogens and facilitate the cell-cycle entrance by the deactivation of retinoblastoma protein (pRB), which is implicated in transcriptional regulation in mammalian cells. In contrast, complexes composed of CDK1/CDK2/cyclin A are necessary for the progression of the cell cycle into the S phase. CDKs in a phosphorylated state are unable to fulfill their function, which leads to cell-cycle arrest [77,90,91]. Moreover, besides the downstream role of CDKs in DDR, these kinases control the activity of the upstream components and target RPA, ATRIP, MDC-1, NBS1, ATM, and CHK1 for phosphorylation, as previously reviewed by our group [77]. Moreover, CHK2 phosphorylates TP53, a protein regarded as the guardian of the genome, which controls the expression of proapoptotic genes (including proapoptotic protein BAX) and inhibitors of antiapoptotic proteins (including phorbol-12-myristate-13-acetate-induced protein 1 (NOXA) and isoform 1 of Bcl-2-binding component 3, isoforms 1/2 (PUMA)), contributing to apoptosis induction, or cyclin-dependent kinase inhibitors (including P21), conferring cell-cycle arrest [78,92].

**Figure 1:**
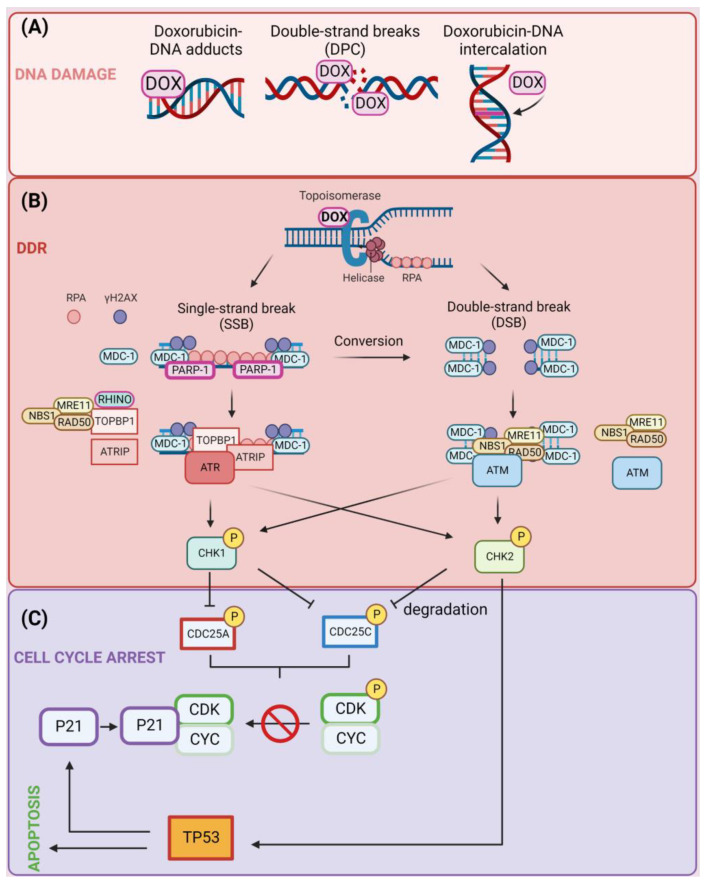
(**A**) Induction of DNA damage following treatment of cells with doxorubicin (DOX). DOX induces DNA damage through three main mechanisms: the formation of DNA adducts, single-strand break (SSB) induction, and double-strand break (DSB) induction, in which DNA strands remain bound to trapped topoisomerase enzymes through DNA–protein crosslinks (DPCs) and intercalation of DOX in the DNA molecule. (**B**) Activation of DNA damage response (DDR) pathway. Trapping of topoisomerase enzymes by DOX induces the formation of either SSBs or DSBs. SSBs are detected by poly [ADP-ribose] polymerase 1 (PARP-1), and the ssDNA is covered with replication protein A (RPA). This results in the recruitment of a variety of factors including DNA topoisomerase 2-binding protein 1 (TOPBP-1), RAD9, RAD1, HUS1 interacting nuclear orphan (RHINO), ATR-interacting protein (ATRIP), MRE11–RAD50–NBS1 complexes, and serine/threonine-protein kinase ATR that contribute to the formation of γH2AX protein foci at the sites of damage. DSBs are detected by MRN complexes, which recruit a mediator of DNA damage checkpoint protein 1 (MDC-1) and amplification of the foci formation by ataxia telangiectasia mutated (A-T mutated) (ATM) kinase. Consequently, ATR and ATM kinases phosphorylate checkpoint kinases CHK1 and CHK2. (**C**) Induction of apoptosis or cell-cycle arrest by DOX. The CHK1/2-mediated phosphorylation of CDC25A/C phosphatases leads to their degradation and as a consequence no dephosphorylation of CDK-cyclin complexes that prevent cell-cycle progression. Alternatively, CHK2 phosphorylates and activates cellular tumor antigen p53 (TP53) transcription factor leading to up-regulation of P21, which binds unphosphorylated (active) CDK–cyclin complexes and contributes to cell-cycle arrest. Created with BioRender.com accessed on 1 January 2023.

Treatment of cells with DOX induces ATM autophosphorylation on Ser-1981, its activation, and ATM-mediated phosphorylation of multiple targets, including NBS1, CHK1, and CHK2. Ascorbic acid treatment does not affect DOX-induced TP53 phosphorylation and accumulation, while preincubation of cells with the hydroxyl radical scavenger N-acetylcysteine attenuates DOX-mediated phosphorylation of TP53, H2AX, NBS1, CHK1, and CHK2. This suggests that hydroxyl radicals contribute to DOX-induced activation of ATM-dependent pathways [93]. Moreover, activation of CHK2 in DOX-treated cells may be independent of ATM or ATR activation [94]. In acute lymphoblastic leukemia, DOX treatment results in cell-cycle arrest in the G2/M phase of the cell cycle via activation of the ATR-CHK1 pathway. The combination of ATR-CHK1 inhibition with DOX treatment results in the synergistic cytotoxic activity that is associated with aberrant chromosome segregation and mitotic spindle defects in cells [95]. Furthermore, inhibition of the DDR pathway components including ATM [96], ATR [97], CHK1/2 [98,99,100], DNA-PKcs [101], and PARP-1 [102] sensitizes cancer cells to DOX treatment. DNA damage caused by topoisomerase II inhibition leads to a G1 and G2 cell-cycle arrest and eventual induction of the apoptosis process [103,104,105,106]. The induction of DNA damage (A), DDR (B), and the contribution of these events to apoptosis or cell-cycle arrest (C) are presented in Figure 1.

### 2.2. Apoptosis–ROS Interplay

Reactive oxygen species (ROS) can be generated in aerobic organisms through the electron transport chain (ETC), the action of catabolic oxidases, and the metabolism of peroxisomes [107]. ROS normally function as cellular messengers in redox signaling events when their concentrations are kept relatively low [108]. Nevertheless, an excessive generation of ROS can contribute to DNA damage through the action of radicals on DNA bases and the sugar–phosphate backbone [109,110]. Unrepaired damage can lead to apoptosis [111,112], cell-cycle arrest [113,114], and senescence [115,116,117,118].

DOX binds directly to cardiolipin on the inner mitochondrial membrane, where it initiates a process of ROS production [119,120,121]. High amounts of ROS cause substantial damage to the mitochondrial structure, which ultimately results in cell apoptosis [122]. In cardiac cells, mitochondria-derived ROS and calcium play an important role in the stimulation of DOX-induced intrinsic and extrinsic forms of apoptosis. This stimulation occurs through the nuclear factor of activated T-lymphocytes (NFAT)-mediated FAS antigen ligand (FASL) up-regulation and down-regulation of FLICE/caspase-8 inhibitory protein (FLIP) [123]. In a study evaluating DOX’s influence on death ligands in induced-pluripotent-stem-cell-derived cardiomyocytes, death receptors including tumor necrosis factor receptor 1 (TNFR1), FAS, and death receptor 5 (DR5) were up-regulated and apoptosis was exacerbated by TNF-related apoptosis-inducing ligand (TRAIL) [124,125]. DOX’s severe cardiotoxicity [126,127] and thrombocytopenia [128] side effects severely restrict its therapeutic utility. DOX induces cardiotoxicity via multiple routes [129,130,131,132], as previously reviewed by multiple authors [7,9,133,134,135]; however, excessive production of ROS and reactive nitrogen species (RNS) constitutes the main mechanism of cardiotoxicity of anthracyclines [136].

The ATM can be also activated by ROS without the involvement of MRN complexes and in the absence of DSBs, as was determined in cells exposed to hydrogen peroxide [137]. The activation of ATM as a consequence of ROS accumulation does not involve the classical DDR response. Some enzymes, such as peroxiredoxin-2 (PRDX2) and thioredoxin-1 (TRX1), can chemically alter cysteine (Cys-2991) residues on ATM when the level of ROS reaches a certain threshold. This leads to the generation of intermolecular disulfide bonds and the activation of ATM dimers. When this disulfide bond formation is prevented (by ATM mutation), the amount of ATM that is activated by ROS is decreased, while the activation route mediated by the MRN complex appears to be unaffected. The notion that ROS are capable of activating ATM independently on MRN complexes is supported by the finding that the activation of ATM by ROS can be impeded by the addition of a reducing agent [137,138,139]. ATM dimers generated in response to ROS exposure still retain their function and can contribute to the phosphorylation of CHK2 and as a consequence activation of the apoptosis pathway [138].

The mechanisms of DOX-induced apoptosis as well as the involvement of ROS in apoptotic signaling were evaluated in osteosarcoma Saos-2 cells. A 48 h treatment of cells with DOX resulted in the accumulation of cells in the pre-G1 phase and induction of DNA laddering, both of which are characteristic features of apoptosis. Additionally, DOX caused an increase in the generation of hydrogen peroxide and superoxide followed by depolarization of the mitochondrial membrane, the release of cytochrome c, and the activation of the caspase-3 enzyme. Furthermore, the DOX treatment caused an increase in the levels of proapoptotic BAX protein and a decrease in the levels of antiapoptotic BCL-2 protein. The fact that catalase was able to inhibit DOX-induced ROS generation, mitochondrial cytochrome c release, procaspase-3 cleavage, and apoptosis in Saos-2 cells provided further evidence that ROS play a role in DOX-induced cancer-cell death. The findings of the above-mentioned research revealed that ROS might function as the signal molecules for DOX-induced cell death, and the mechanism may be functional despite the absence of TP53 [140]. Similar results were obtained in MCF-7 breast cancer cells, where treatment with DOX induced BAX protein expression with concomitant caspase-8 and caspase-3 elevation and down-regulation of BCL-2 protein. Moreover, the treatment with the chemotherapeutic agent increased the generation of hydrogen peroxide with a decrease in nuclear factor kappa-light-chain-enhancer of activated B cells (NF-κB) expression. In contrast, the increase in superoxide dismutase (SOD2) expression contributed to the diminishment of hydrogen peroxide production with a decrease in NF-κB protein expression in the MDA-MB-231 breast cancer cell line [141]. It is hypothesized that the greater amounts of superoxide that are detected during DOX administration are caused by a decrease in the activity of enzymes that are responsible for the decomposition of superoxide, such as SOD2 and catalases [142]. Treatment of cervical cancer cells (CaSki and SiHa cell lines) with cisplatin and DOX results in different profiles of types of ROS induced following drug administration. Cisplatin treatment results in the generation of hydrogen peroxide, hydroxyl, and peroxyl radicals, while DOX induces the production of superoxides [143]. A similar tendency was observed in HaCaT keratinocytes, where DOX induced superoxide production with no increase in hydrogen peroxide, hydroxyl, or peroxyl radicals. This study also pointed out that the diminished antiapoptotic function of the BCL-2 protein may result from its ubiquitination and proteasomal degradation [144]. Alternatively, down-regulation of GATA binding protein 4 (GATA4) can result in a decrease in antiapoptotic B-cell lymphoma-extra large (BCL-xL) protein [145]. Ehrlich tumor cells treated with DOX experience excessive superoxide anion production in tumor microsomes and nuclei. Rotenone (ETC complex I interferer) was shown to boost the formation of drug-stimulated superoxide by mitochondria. This suggests that the proximal section of the ETC in tumor cells is responsible for the reduction of the DOX quinone. These studies also show that anthracyclines are capable of considerably boosting oxygen radical metabolism in Ehrlich tumor cells and this may contribute to the cytotoxicity and apoptotic properties of agents belonging to this class of compounds [146].

Lipid peroxidation, the oxidative destruction of lipid membranes, has been linked to oxidative stress. This procedure generates about 200 different kinds of aldehydes, many of which are highly reactive. The mutagenic and carcinogenic effects of these aldehydes are due to their capacity to react with DNA or to form pro-mutagenic exocyclic adducts and protein–DNA crosslinks, which can halt both DNA replication and transcription [147,148,149]. Lipid peroxidation was observed following DOX administration [150,151,152]; however, the contribution of lipid peroxidation to DOX cytotoxicity is controversial, as previously reviewed [153].

Another possible mode of action of DOX includes the overproduction of ceramide [154,155], which contribute to ROS generation, DNA damage, and apoptosis [6,129,156,157,158,159]. Ceramides were reported to trigger the release of proapoptotic proteins from the mitochondria through the development of vast protein-permeable channels through which proapoptotic proteins (including cytochrome c, procaspases, apoptosis-inducing factor (AIF), heat shock proteins, the secondary mitochondria-derived activator of caspases (SMAC)/direct inhibitor of apoptosis-binding protein with low pI (DIABLO), and endonuclease G) are released. Mitochondria contain the enzymes necessary for the synthesis and hydrolysis of ceramide. There is also evidence that mitochondrial ceramide levels are increased just before the induction phase of apoptosis [160,161,162]. Khodadust et al. found that a dendrimeric (PIC-G4-DOX) nanoformulation of DOX up-regulates the expression of genes encoding the proapoptotic proteins NOXA, PUMA, and BAX, while down-regulating the expression of survivin, APOLLON, and BCL-2 in the MCF-7 cancer cell line. This formulation also increased the cytotoxic activity of DOX toward the MCF-7 cancer cell line. According to the findings, PIC-G4-DOX has the potential to be effective for targeted delivery that can impact apoptotic pathways, which ultimately leads to a higher degree of cancer-cell killing. The Polyinosinic:polycytidylic acid [Poly (I:C)] component of the PIC-G4-DOX nanoformulation acts similarly to an RNA virus and triggers the intrinsic apoptosis pathway of cancer cells. Following receptor-mediated endocytosis, the synthetic double-stranded RNA is detected in the cytoplasm by a class of ubiquitous cytoplasmic RNA helicases called retinoic acid inducible gene-I (RIG-I) and melanoma differentiation Ag-5 (MDA-5) initiates an antiviral signaling cascade via the adaptor protein mitochondrial antiviral signaling (MAVS). The activation of TP53 as a result of stress stimuli leads to the activation of BAX, which in turn activates the intrinsic apoptotic pathway. BAX achieves this activation via up-regulating PUMA and NOXA. After being activated, proapoptotic BAX causes the mitochondria to become depolarized, which in turn causes the release of cytochrome c into the cytoplasm. After forming an apoptosome by creating a complex with apoptosis protease activation factor (APAF-1), cytochrome c and pro-caspase 9 lead to caspase 9 activation, which in subsequent events activates effector caspase 3. The activation of the transcription factor nuclear factor kappa B (NF-kB) and interferon regulatory factors (IRFs) such as IRF-3 as a result of MAVS-dependent signaling triggers the production of inflammatory cytokines and chemokines, including type I interferons (IFNs). Activation of RIG-I and subsequent downstream MAVS signaling can induce transcription of the proapoptotic BH3-only proteins NOXA and PUMA independent of TP53. This results in the activation of the intrinsic apoptotic pathway, in which BAX functions to disrupt the integrity of the mitochondrial membrane, which in turn leads to the release of cytochrome c, formation of an apoptosome, caspase 3 activation, and apoptosis. Because of its stability, biocompatibility, increased rate of internalization, and toxicity, PIC-G4-DOX shows promise as potential anticancer agent [163].

**Figure 2:**
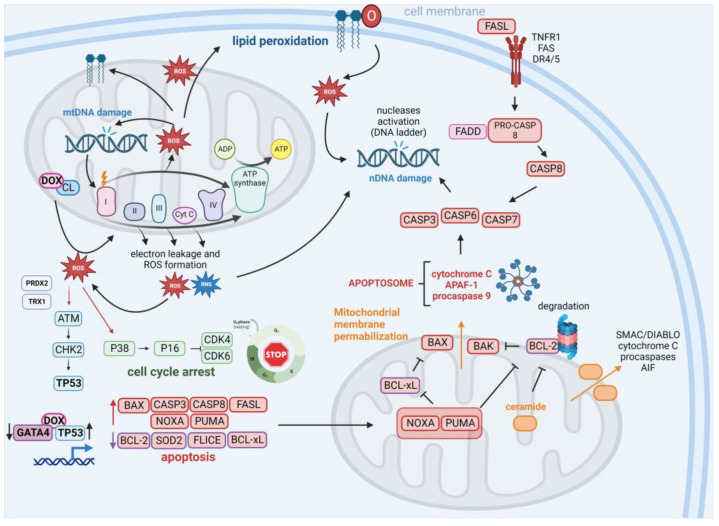
Contribution of DOX to ROS induction and apoptosis. The interaction of DOX with cardiolipin (CL) leads to the elevation of reactive oxygen species (ROS) and reactive nitrogen species (RNS) levels, which contribute to DNA damage of nuclear (nDNA) and mitochondrial DNA (mtDNA). DNA damage and mutations in mtDNA encoding proteins of the electron transport chain (ETC) lead to the production of defective ETC components that increase electron leakage and ROS/RNS generation. Alternatively, ROS trigger lipid peroxidation, enhancing ROS pools in the cells. ROS may trigger the activation of the ATM-CHK2-TP53 signaling independently of DNA damage through ROS-mediated ataxia telangiectasia mutated A-T mutated (ATM) dimer formation through modification of enzyme cysteine residues by peroxiredoxin-2 (PRDX2) and thioredoxin-1 (TRX1). Through the P38 protein, ROS may activate the P16 protein, which works as an inhibitor of cyclin-dependent kinases 4/6 (CDK4/CDK6). DOX increases the expression of cellular tumor antigen p53 (TP53) and down-regulates the expression of GATA binding protein 4 (GATA4), which influences the expression of genes under the control of these transcription factors. Consequently, an increase in multiple proapoptotic proteins (including BAX, caspase 3/8 (CASP3/8), FAS antigen ligand (FASL), phorbol-12-myristate-13-acetate-induced protein 1 (NOXA), and isoform 1 of Bcl-2-binding component 3, isoforms 1/2 (PUMA)) is observed in addition to a decrease in antiapoptotic factors (such as cyclin D1, B-cell lymphoma-extra large (BCL-xL), and FLICE). The observed down-regulation of BCL-xL and apoptosis regulator Bcl-2 (BCL-2) activity can be attributed to the inhibitory activity of NOXA and PUMA and the antiapoptotic protein proteasomal degradation. Alternatively, activation of ceramide signaling together with mitochondrial pore formation (through BAX and BAK proteins) and the loss of mitochondrial integrity confer the release of proapoptotic factors including cytochrome c, apoptotic protease-activating factor 1 (APAF1), and procaspase-9 (leading to apoptosome formation), the secondary mitochondria-derived activator of caspases (SMAC)/direct inhibitor of apoptosis-binding protein with low pI (DIABLO), apoptosis-inducing factor 1, mitochondrial (AIF), and pro-caspases. Apoptosome activity contributes to the activation of effector caspases (CASP3, -6, and -7) that execute the final phase of apoptosis and lead to the activation of nucleases that cleave nDNA (leading to DNA laddering observed following treatment of cells with DOX). DOX also induces the extrinsic apoptosis pathway via up-regulation of FASL, its interaction with the death receptors tumor necrosis factor receptor 1 (TNFR1), FAS, and death receptor 5 (DR5), and activation of CASP8 and effector caspases. Created with BioRender.com accessed on 1 January 2023.

Inconsistency exists on the role of TP53 in DOX-induced apoptosis. While some studies confirm the participation of TP53 in this process, others seem to contradict these finding. Discrepancies in the methodologies utilized for the exploration of TP53’s role in DOX-mediated apoptosis and heterogeneity of the tumors that were evaluated in these studies could be accountable for these conflicting results, as previously reviewed [153]. The contribution of DOX to ROS induction and apoptosis is presented in Figure 2.

### 2.3. Senescence Induction

It is well known that cancer cells treated with chemotherapeutics can enter senescence. As a result, therapy-induced senescence (TIS) has emerged as an advantageous method for combating cancer with minimal side effects. Emerging evidence suggests that the senescence of cancer cells can be undesirable due to the propensity of senescent cells to establish a pro-cancerogenic milieu. To deal with senescent cells, researchers have created a new drug class called senolytics, which specifically remove senescent cells [164].

The characteristic features of senescent cells include cell-cycle arrest, expression of senescence-associated β-galactosidase, heterochromatin foci formation, telomere shortening, hyper-methylation of histone H3K9, and secretion of multiple factors including chemokines and inflammatory factors such as MMPs, IL-1, IL-6, and IL-8 (the so-called senescence-associated secretory phenotype; SASP) [165]. This topic was recently reviewed by multiple authors [166,167].

DOX-induced senescence was established as the primary mechanism of therapeutic activity of the drug in the FU-SY-1 synovial sarcoma cell line [168]. Bojko et al. studied the effects of DOX (concentration of 100–200 nM) on different cancer-cell lines (non-small-cell lung cancer (A549), neuroblastoma (SH-SY-5Y), colon (HCT116), and breast cancer (MDA-MB-231 and MCF-7)). Growth arrest, induction of DSBs, up-regulation of P21, and ATM activation (the latter was not activated in HCT116 cells) was shown following drug treatment [164]. This is consistent with the earlier finding on DDR activation and senescence induction in cells treated with DOX [169]. Moreover, NF-κB activation in response to the activity of ATM could contribute to the development of SASP in DOX-treated cells. Furthermore, the induction of senescence in the examined cells was found to be dependent on the cell line used in the experiment [170,171]. As opposed to ATM and DNA-PKsc, ATR kinase activity cannot be entirely replaced by other PIKKs, as demonstrated in previous experiments by the same authors, making it essential for the development of senescence. Furthermore, the authors demonstrated that knocking down the NF-κB component, P65, abolished SASP induction but did not affect other markers of senescence and DDR signaling, demonstrating that SASP is independently controlled by NF-κB activated in other ways than the classical PIKK signaling cascade [169]. Furthermore, the same team evaluated the senescence induction in HCT116 cells treated with low doses of DOX. Increased expression of TP53, P21, and cyclin D1 accompanied by polyploidization through endoreduplication was found following the drug treatment [172].

Intimate cross-talk between senescence, epithelial–mesenchymal transition (EMT), and autophagy exist. Hu et al. observed time-dependent DOX-induced senescence activation in HeLa cells accompanied by elevated expression of IL-6 and IL-8. Due to DOX’s ability to trigger EMT, cancer cells acquire enhanced motility and resistance to chemotherapy (as the expression of pro-inflammatory IL-8 and CDK inhibitory protein p16^INK4a^ were inversely related to that observed for vimentin). This study, therefore, indicated that EMT could suppress senescence induced by DOX [165]. This is consistent with the finding that DOX-induced senescence encourages stemness and tumorigenicity in a hepatocellular carcinoma cell line (HuH-7) [173] or induces autophagy in the myelogenous leukemia cell line (K562) [174].

Multiple other studies have also shown that DOX may also trigger senescence in normal cells, including fibroblasts [175], microglia cells [176], cardiac progenitor cells [177], cardiomyocytes [178,179], mesenchymal stem cells [180], and thymus cells [181]. This should be taken into account when investigating the potential anti-tumor activity of DOX related to senescence induction.

### 2.4. Other Types of Cell Death

Autophagy is a cellular catabolic degradation response that occurs in response to cell famine or stress. This reaction involves the engulfment, digestion, and recycling of cellular proteins, organelles, and cytoplasm to maintain cellular metabolism. The process of autophagy has a dualistic role in cancer. Catabolism through autophagy is beneficial to cellular survival. However, an imbalance in cell metabolism, which occurs when autophagic cellular consumption surpasses the cellular capacity for synthesis, encourages the death of cells. This can be partially explained by the encouragement of necrotic cell death and an inflammatory response that occurs in tumors that have abnormalities in both autophagy and apoptosis. Loss of the pro-survival role of autophagy is known to promote the development of tumors [182]. Autophagy was found to hamper DOX-induced apoptosis in osteosarcoma [183] and confer the chemoresistance to DOX in MCF-7 cells [184]. The induction of autophagy by rapamycin in the presence of DOX increases cardiac cell viability, decreases apoptosis and ROS production, and refines mitochondrial function in vitro and in vivo [185]. In contrast, inhibition of autophagy escalates the cytotoxicity of DOX in MDA-MB-231 and MCF-7 breast cancer cells [186,187] and prostate cancer cells [188].

Ferroptosis was indicated as another mechanism of DOX-induced cardiotoxicity. DOX causes an increase in the cell’s labile iron pool, which contributes to its cytotoxicity. DOX and its metabolites have the potential to disrupt iron homeostasis through their ability to inactivate iron regulatory proteins 1 and 2 (IRP1 and IRP2). The expression of genes involved in iron metabolism can be altered as a result of the binding of inactive IRPs to iron-response elements (IREs). By inhibition of GPX4 in both the cytosol and the mitochondria, DOX causes lipid peroxidation, which in turn results in ferroptosis. Alternatively, DOX contributes to the activation of nuclear factor erythroid 2-related factor 2 (NRF-2), which elicits ferroptosis. This topic was previously reviewed [189]. In line with these findings, nanoparticles consisting of DOX and exogenous ferritin were developed. The nanoparticles induced ROS formation and ferroptosis of HT-29 cells [190]. Similarly, other formulations including microenvironment-activatable Fe–DOX preloaded amorphous CaCO_3_ nanoparticles [191] and iron–DOX prodrug-loaded liposomes [192,193] were developed. Induction of ferroptosis, a relatively newly identified mechanism of cell death, can take place as a result of lipid peroxidation that is mediated by the Fenton reaction. The effectiveness of Fenton-reaction-dependent ferroptosis is, however, hindered in tumor cells by low levels of hydrogen peroxide and elevated levels of glutathione [192,194].

Pyroptosis is an inflammatory form of programmed cell death with a dualistic role in cancer. On the one hand, pyroptosis, in its role as an inflammatory cell-death process, creates an environment that is favorable for the growth of tumors. On the other, an over-activation of pyroptosis can limit the growth of tumor cells [195,196]. Caspase-1, -4, -5, and -11 are involved in the execution of pyroptosis. When caspase-1 is activated, the preforms of IL-1β, IL-18, and gasdermin D (GSDMD) are processed into active forms. In contrast to caspase-1, which is triggered by pyroptotic sensors such as the NLR family pyrin domain containing 3 (NLRP3) inflammasome, caspase-4, -5, and -11 are activated when they directly attach to lipopolysaccharides. In the past, it has been noted that NLRP3 is significant with regard to the cardiotoxicity of DOX, as discussed earlier [189,197]. Moreover, DOX caused a significant decrease in mouse muscular function, which was connected with a considerable increase in the NLRP3 inflammasome and the initiation marker toll-like receptor 4 (TLR4). When compared to the controls, there was a considerable rise in the pyroptosis activator known as apoptosis-associated speck-like protein containing a caspase recruitment domain (ASCO), which was accompanied by overexpression of certain pyroptosis markers (caspase-1, IL-1, and IL-18) [198]. When breast cancer cells were treated with DOX, the cell viability was decreased in a dose-dependent manner. Additionally, pyroptosis morphology was elicited in MDA-MB-231 and T47D cells. GSDME seems to be a crucial regulator of DOX-induced pyroptosis. In addition to this, DOX treatments caused an increase in the amount of ROS, activation of the C-Jun N-terminal kinase (JNK) signaling pathway, and activation of caspase-3 [199]. Pyroptosis was triggered by DOX at concentrations ranging from 0.5 to 5 µmol/L in melanoma cell lines SK-MEL-5, SK-MEL-28, and A-375 that had high levels of deafness, autosomal dominant 5, isoform CRA a (DFNA5) expression, but not in the human breast cancer cell line MCF-7, which had low levels of DFNA5 expression. On the other hand, treatment with DOX prompted melanoma cells to begin the process of autophagy and the use of an autophagy inhibitor triggered the pyroptosis of cells. In DOX-treated human melanoma cells, eukaryotic elongation factor-2 kinase (eEF-2K) directs the cross-talk between pyroptosis and autophagy. The absence of eEF-2K results in the inhibition of autophagy and enhancement of pyroptosis, which contributes to the modification of the sensitivity of melanoma cells to DOX. This finding suggests that targeting eEF-2K may enhance the antitumor activity of DOX [200].

## 3. DOX and Tumor–Immune Microenvironment

Through many mechanisms involving coordination between innate and adaptive immune responses, the body can recognize cancer cells and eradicate them. The role of T cells in this process is crucial. When stimulated, these cells contribute to an immune response that targets cancer cells. For T cells to become engaged, a specific peptide epitope of the antigen must be presented on the major histocompatibility complex (MHC) of an APC, and then form a complex with the T-cell receptor on T cells. T-cell activation requires a second signal produced by the binding of co-stimulatory molecules [201]. The process is complex and involves tumor-cell recognition, recruitment of APCs, antigen uptake and its processing, APC maturation, antigen presentation to T lymphocytes, production of stimulatory factors including interferons (IFNs, including IFNγ), and engagement of cytotoxic CD8+ T lymphocytes [202]. Moreover, the coinhibitory receptor PD-1 can be constitutively expressed or activated in myeloid, lymphoid, normal epithelial cells, and cancer. Programmed death ligand 1 (PD-L1) represents the main ligand of the PD-1 receptor. The PD-1/PD-L1 association is crucial in the establishment of immunological tolerance under normal conditions, where it serves to suppress excessive immune cell activity that could otherwise cause tissue damage and autoimmune response. Cancer cells, however, utilize PD-L1 to avoid detection by the immune response [203].

Conventional chemotherapy’s impact on the immune system is becoming increasingly recognized. This is because most studies evaluating the efficacy of such drugs against cancer cells are conducted in vitro or on immune-compromised animals and do not include immunological follow-ups [204]. DOX not only exhibits direct cytotoxic effects on cancer cells, but also utilizes the immune system to kill cancer cells by triggering CD8^+^ T-cell responses [205]. Moreover, pre-treatment with DOX in cancer patients may be an effective strategy to boost anti-cancer immune responses by increasing antigen-specific CD4^+^ Th1 immune responses [206,207]. The incubation of cancer cells with DNA-damaging agents, including the topoisomerase inhibitors camptothecin [208], DOX [209,210,211], and irinotecan [212] or the alkylating agents carboplatin [213,214], cisplatin [215,216,217,218,219], and oxaliplatin [220], leads to up-regulation of PD-L1. In response to drugs that cause DSBs, including etoposide or ionizing radiation, the ATM/ATR/CHK1 pathway is activated, which causes PD-L1 expression to increase. In addition, PD-L1 expression levels return to normal in cells that escape death after being exposed to ionizing radiation, suggesting that the up-regulation of this immune checkpoint protein in response to DNA damage is only temporary [221]. In addition, DSBs activate the interferon regulatory factor 1 (IRF1) and signal transducer and activator of transcription 1/3 (STAT1/3) transcription factors. Notably, IRF1 is necessary for PD-L1 overexpression, which shows that the DSB-dependent up-regulation of PD-L1 is regulated through the traditional JAK1/2-STAT1/2/3-IRF1 pathway [221,222,223,224,225].

DOX treatment induces the up-regulation of immune-related genes of the PD-1/PD-L1 and T-cell cytotoxicity pathways. This is accompanied by the induction of the tyrosine-protein kinase JAK (JAK-STAT) pathway and tumor necrosis factor α (TNF-α) signaling in triple-negative breast cancer patients [210] and can lead to up-regulation of PD-L1 in cancer cells [209]. PD-1/PD-L1 axis blockage together with DOX treatment may help to overcome resistance to DOX treatment and provide clinical benefits for patients [210,226]. It is possible that the up-regulation of PD-L1 in cancer cells could increase the availability of epitopes to which anti-PD-L1 small-molecule compounds and antibodies could bind. This could be one of the explanations for the observed enhanced therapeutic efficacy of the combined treatment of DOX and ant-PD-L1 antibodies [227,228]. In contrast, PD-L1 up-regulation can also confer chemiresistance in malignant cells through the up-regulation of multidrug resistance 1/P-glycoprotein (MDR1/P-GP) in breast cancer cells [229]. Therefore PD-1/PD-L1 inhibition could help to overcome the resistance mechanism to commonly used chemotherapeutic agents [230].

However, the difficulty lies in combining chemotherapeutic medications with anti-PD-L1 compounds to increase the effectiveness of the combined treatment beyond that of the individual treatments and reduce the negative effects of the therapies. A combination of the chemotherapeutic agent with the anti-PD-L1 agent should be chosen not only for its capacity to effectively kill cancer cells and inhibit their growth but also based on its predisposition to modulate the activity of immune-active cells and to preserve the activity of the administered immunotherapy [231]. For instance, PD-L1 expression is increased in bone marrow stromal cells or osteosarcoma cell lines and clinical tissue samples obtained following DOX treatment. DOX prevents CD8^+^ T-lymphocyte proliferation and also increases the rate of immune-cell apoptosis. The use of an antibody against PD-L1 can revert these diminishing effects on the anti-tumor immune response and lead to elevated tumor-cell apoptosis [211,232]. Pembrolizumab and atezolizumab are examples of two anti-PD-L1 monoclonal antibodies that are frequently paired with DOX to treat a variety of malignancies including triple-negative breast cancer (TNBC). A recent clinical investigation showed a significant elevation of immune-related genes involved in the PD-1/PD-L1 pathway and T-cell cytotoxicity pathways in TNBC patients treated with DOX and cisplatin [210].

Over the years, numerous systems and strategies have been tested for their effectiveness in directing drugs toward tumors. Multiple systems have been developed, including liposomes, polymers, micelles, nanoparticles, and antibody conjugates [233]. The accumulation of antitumor drugs in tumors is enhanced, and the solubility, stability, and bioavailability problems often associated with these agents are avoided through the nanoformulation of drugs into liposomes [234]. The introduction of the liposomal drug-delivery method has had a profound impact on the pharmaceutical industry. Liposomes are synthetic vehicles that can be made using cholesterol and natural, non-toxic phospholipids to generate a spherical vesicle. Liposomes constitute a promising technology for drug delivery because of their size and hydrophobic and hydrophilic properties, which hallow the encapsulation of drugs, low toxicity, and biocompatibility. Passive drug loading occurs when the drug is contained in the liposome during preparation, while active drug loading occurs after the formation of the liposome [235,236].

In 1995, the FDA approved the first liposomal DOX, called Doxil^®^ (injectable DOX hydrochloride liposomes), for the treatment of ovarian cancer, Kaposi’s sarcoma, and myeloid melanoma [236]. The PEGylation of liposomes significantly contributed to the advancement of Doxil in clinical trials. PEGylation refers to the covalent attachment of polyethylene glycol (PEG) to a given molecule. It provides several benefits in drug development: it prolongs the retention time of liposomes in the body and shields them from degradation by intracellular enzymes or other mechanisms active inside the cell [237,238]. Pre-formulation and post-formulation techniques are distinguished as types of liposomal surface modification. Before formulation, phospholipids (often PEGylated) undergo covalent-bond formation with ligands. After conventional lipids are combined with ligand-anchored lipids, the active drug is loaded into the liposomes remotely. Through the addition of transferrin to the distal ends of liposomal PEG chains, Eavarone et al. designed liposomes containing DOX selectively targeting glioma C6 cells expressing transferrin receptor. This formulation exceeded the uptake efficiency of non-PEG, PEG, and albumin-coupled PEG liposome drug-delivery systems [239]. Targeted liposomal formulations constitute the next generation of liposomal drug-delivery systems and have been created to increase the selectivity of liposomes for cancerous tissues. Considering their great specificity for their target antigens, antibodies and antibody fragments are the most popular targeting moieties for liposomes. Interest in antibody-modified liposomes for the targeted delivery of anticancer drugs is high [240]. Effective treatment of B-cell cancers may involve the monoclonal-antibody-mediated delivery of liposomal anticancer medicines to surface antigens expressed on malignant B cells [241]. Liposomal agents targeting the internalizing epitopes (such as CD19) are hypothesized to be more effective than those targeting non-internalizing epitopes (e.g., targeted against CD20). When comparing DOX-loaded anti-CD20 targeted liposomes and nontargeted liposomes, DOX-loaded anti-CD19 targeted liposomes dramatically increased survival times after a single i.v. dosage. It has been thereby empirically established that internalizing epitopes are more beneficial to target therapeutically than their non-internalizing counterparts [242,243]. Studies on CD22 suggest a multifaceted function for this transmembrane glycoprotein in controlling B-cell survival and proliferation [244]. Through the conjugation of HB22.7 anti-CD22 monoclonal antibody with PEGylated liposomal DOX, an immunoliposomal formulation with increased efficacy and without increased toxicity was generated [245,246].

The FDA’s green light for Doxil^®^ led the way for more liposomal formulations to be used in clinical settings. In addition to Doxil^®^, another licensed liposomal formulation is Myocet^®^, which provides the same benefits as Doxil^®^ without displaying hand–foot syndrome (palmar–plantar erythrodysesthesia syndrome), a prominent side effect of pegylated liposomal DOX formulations [247,248,249,250,251]. The generic DOX hydrochloride liposomal injectable Lipodox^®^ has been approved for use in patients [252,253,254]. Studies on other DOX liposomal preparations including Caelyx^®^ [255,256], Thermodox^®^ [257,258], Nudoxa^®^ [259,260], 2B3-101 [261,262,263], and C225-ILS-Dox [264] with unique anticancer efficiencies, tissue distributions, and toxicity profiles have also been conducted. This topic was extensively reviewed by other authors [153,265,266]

Some of the formulations were tested for their beneficial effect on the tumor-directed immune response. For example, chimeric polypeptide DOX nanoparticle formulation boosted the infiltration of leukocytes into the tumor, reducing tumor-cell growth and inhibiting the metastatic potential of 4T1 mammary carcinoma cells of BALB/c mice compared with conventional DOX. These findings demonstrate that a nanoparticle drug is distinct from the free drug in its ability to activate antitumor immunity depending on CD8^+^ T-cell stimulation and IFN-γ production [267]. In addition, the number of immunosuppressive tumor-associated macrophages (TAMs) in the tumor bed is reduced by DOX treatment. STAT1-deficient mice, on the other hand, show decreased T-cell activation and reduced T-cell infiltration of the tumor in response to drug treatment and show resistance to lapatinib and/or DOX’s effects on tumor growth. Additionally, STAT1 deficiency leads to decreased production of C-X-C motif chemokine receptors (CXCL9, CXCL10, and CXCL11) in the tumor epithelium, all of which work as T-cell chemotactic factors, suggesting STAT is an important contributor to DOX’s effects on the antitumor immune response [268]. Increased numbers of CD8^+^ T lymphocytes and suppression of metastasis are observed in dendritic-cell infusion and DOX treatment of mice with metastatic tumors. DOX-treated mice exhibit higher levels of calreticulin (CRT). CRT is a protein that resides in the endoplasmic reticulum (ER), and it plays an important role in the regulation of calcium homeostasis as well as glycoprotein folding in the ER. Additionally, the protein has been found on the cell surface of necrotic and apoptotic cells, where it is thought to play a part in immunogenic cell death as well as other extracellular functions. Following an exposition on the apoptotic cell, CRT interacts with the CD91 receptor on DCs, playing an important role in cancer-cell recognition by the immune system [269,270]. Additionally, high mobility group box protein B1 (HMGB1) expression is also increased following the treatment of cells with DOX [271]. HMGB1 is a nuclear molecule that is typically secreted from necrotic cells. It is believed that HMGB1 is involved in the structural organization of DNA. After being released into the extracellular space, HMGB1 acts as a mediator of the inflammatory response. This could enhance the anti-tumor immune response. Apoptotic cells, on the other hand, typically do not release HMGB1 and are “immunologically silent”, in contrast to necrotic cells [271,272]. Upon the administration of DOX, metastatic human melanoma SK-MEL-24 and human colon cancer LS174T cells undergo senescence; however, the less-metastatic cell lines SK-MEL-28 and DLD-1 prefer apoptosis following drug treatment. The expression of HMGB1 was found to be persistent in senescent B16-F10, SK-MEL-24, and LS174T cells that had been treated with DOX. This led to a rise in P21 levels. Furthermore, HMGB1 depletion caused a shift from senescence to apoptosis in B16-F10 cells, and HMGB1 overexpression managed to trigger the transition from apoptosis to senescence simultaneously with increased P21 expression in B16 cells after treatment with DOX. According to these findings, selective induction of tumor suppression might be possible through the use of HMGB1 regulation of malignancies that have distinct metastatic capability [273]. HMGB1, which is released by cancer cells, serves as a ligand for the TLR-4 receptor on DCs. Sera IFNγ levels are highest after treatment with DCs and DOX, which contributes to the induction of immunological cell death [270,273].

Yang et al. generated DOX prodrug nanoparticles (CAP-NPs) via conjugation of the cathepsin B-cleavable peptide with DOX that allowed the release of the DOX only in cancer cells over-expressing cathepsin B. Moreover, when compared with the treatment of conventional free DOX with anti-PD-L1 antibodies, the combinatorial therapy of CAP-NPs with the same immune-checkpoint inhibitor demonstrated a significantly higher rate of complete tumor regression (50%) than the latter. During this time, CAP-NP-treated mice experienced significantly fewer adverse effects that were associated with DOX treatment and PD-1/PD-L1 blockage [34,274]. It has been also demonstrated that PD-L1-directed liposomes encompassing DOX are a reasonable combination that is capable of improving the activity of the immune system by blocking PD-L1 and through discriminate internalization of DOX in cancer cells. This combination has been effective in providing a dual benefit that is achieved by both the chemo- and immune-therapeutic strategies [275].

**Figure 3:**
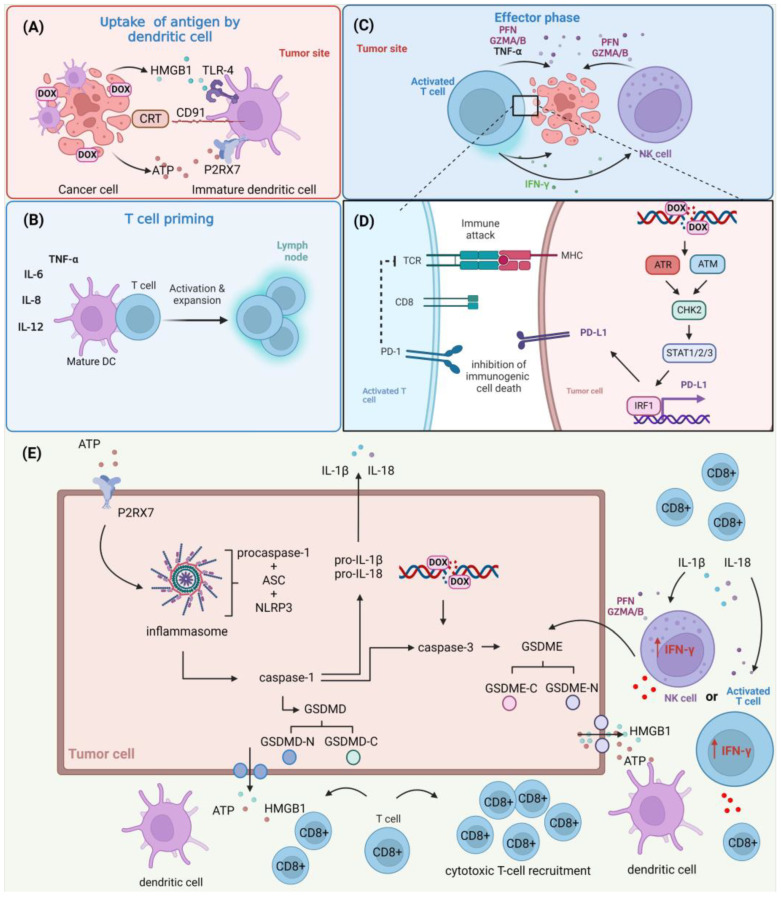
The role of DOX in the anti-tumor immune response. (**A**) DOX induces the apoptotic cell death of cancer cells, contributing to the exposition or release of ATP, calreticulin (CRT), and high mobility group box protein B1 (HMGB1) of cancer cells and promoting their recognition by dendritic cells (DCs) through respective receptors (P2X purinoceptor 7 (P2RX7), CD91, and toll-like receptor 4 (TLR-4)) present on their cellular surface. (**B**) These events allow the maturation of DCs and the release of immune-stimulating factors such as interleukins (ILs) and tumor-necrosis factor α (TNF-α). These events promote the activation and expansion of T cells. (**C**) Activated T cells and natural killer (NK) cells recruited to the tumor microenvironment release perforins (PFNs), granzyme A/B (GZMA/B), and interferon γ (IFN-γ), leading to cancer-cell death. (**D**) DOX induces double-strand break (DSB) formation, contributing to the activation of the ATR/ATM/CHK2 pathway and promoting STAT1/2/3/IRF1-mediated programmed cell death 1 ligand 1 (PD-L1) up-regulation. This confers to the inhibition of immunogenic cell death. (**E**) ATP released by dying cancer cells activates P2RX7 expressed on surrounding tumor cells, contributing to the formation of the inflammasome (consisting of adapter protein apoptosis-associated speck-like protein containing a CARD (ASC), NACHT, LRR, and PYD domains-containing protein 3 (NLRP3) and procaspase-1) and activation of caspase-1, which converts pro-IL-1β and pro-IL-18 to active forms (IL-1β, IL-18), activates effector caspase-3, and cleaves gasdermin-D/E (GSDMD/E) to the C-terminal moiety (GSDMD/E-C) and promotes the release of the N-terminal moiety (GSDMD/E-N) that forms pores, triggering pyroptosis. This contributes to the release of a multitude of intracellular factors including ATP, HMGB1, IL-1β, and IL-8 to the extracellular space. Alternatively, the cleavage of GSDMD/E can be triggered by GZNMA/B and PFNs released by NK cells and activated T cells recruited to the tumor microenvironment. Created with BioRender.com accessed on 1 January 2023.

Unlike apoptotic cells, which remain immunologically quiet, pyroptotic cells rapidly leak their cellular contents, such as cell antigens, proinflammatory cytokines, and damage-associated molecular patterns (DAMPs), which can then affect the tumor immune microenvironment, activate host antitumor immunity, and ultimately result in tumor regression. Furthermore, APCs adopt a “hyperactive” state characterized by increased motility and promotion of higher cytotoxic T-lymphocyte responses after being activated by pyroptosis-induced signals [276]. The influence of DOX on the anti-tumor immune response is shown in Figure 3.

## 4. Conclusions

Because of its efficacy in battling a wide spectrum of malignancies, DOX is commonly employed in chemotherapeutic regimens. Despite its widespread clinical use, the mechanisms of action of DOX are still being debated. Although the precise mechanisms of action of DOX remain unknown, it is established that this anticancer agent intercalates into DNA, inhibits topoisomerase enzymes, disrupts the proper functioning of mitochondria, and increases free-radical production and oxidative damage [4]. Besides triggering the mitochondrial apoptosis pathway, DOX also induces senescence, autophagy, pyroptosis, ferroptosis, or necrosis of cancer cells depending on the dose of the drug and the cellular context.

DOX not only has direct cytotoxic effects on cancer cells, but also contributes to the elimination of cancer cells via the activation of immune CD8^+^ T-cell responses. An understanding of the interplay between the DOX-treated cells and the tumor microenvironment seems crucial for the development of combinatorial regimens including immune checkpoint inhibitors such as PD-L1 antibodies and DOX [231].

DOX exerts its effect on normal cells, leading to the development of life-threatening side effects including myelosuppression, nephrotoxicity, and cardiotoxicity [153,189]. Attempts should be made to find more effective ways to manage DOX toxicity. As a result, DOX must be loaded into a special vehicle and delivered to specific tumor sites, minimizing toxicity while maximizing the therapeutic benefits. Therefore, numerous investigations have been conducted on DOX nanoformulations such as liposomes, polymer micelles, dendrimers, and nanogels [277]. Although several DOX nanoformulations have been shown to increase the therapeutic output in comparison to standard preparations, they still face obstacles such as suboptimal diffusion and permeation properties, sophisticated release patterns, immunogenic responses, and undesirable interactions with serum proteins that force their in-depth in vitro and in vivo investigations [4,278].

## Abbreviations

AIF	apoptosis-inducing factor
APAF1	apoptotic protease-activating factor 1
APC	antigen-presenting cell
ASC	adapter protein apoptosis-associated speck-like protein containing a CARD
ASCO	apoptosis-associated speck-like protein containing a caspase recruitment domain
ATM	ataxia telangiectasia mutated (A-T mutated)
ATR	ataxia telangiectasia and Rad3-related
ATRIP	ATR-interacting protein
BCL-2	apoptosis regulator BCL2
BCL-xL	B-cell lymphoma-extra large
CDC25A/C	M-phase inducer phosphatases
CDK	cyclin-dependent kinase
CHK1/2	checkpoint kinases
CL	Cardiolipin
CRT	Calreticulin
CXCL	C-X-C motif chemokine receptors
DAMPs	damage-associated molecular patterns
DDR	DNA damage response
DFNA5	deafness, autosomal dominant 5, isoform CRA
DIABLO	direct inhibitor of apoptosis-binding protein with low
DNA-PKcs	DNA-dependent protein kinase catalytic subunit
DOX	Doxorubicin
DPC	DNA–protein crosslink
DR5	death receptor 5
DSBs	double-strand breaks
eEF-2K	eukaryotic elongation factor-2 kinase
EMT	epithelial–mesenchymal transition
ER	Endoplasmic reticulum
ETAA1	Ewing’s tumor-associated antigen 1
ETC	electron transport chain
FASL	FAS antigen ligand
FDA	Food and Drug Administration
FLIP	FLICE/caspase-8 inhibitory protein
GATA4	GATA Binding Protein 4
GPX4	glutathione peroxidase
GSDMD/E	gasdermin-D/E
GZMA/B	granzyme A/B
HMGB1	high mobility group box protein B1
HR	homologous recombination
IFNs	Interferons
ILs	Interleukins
IREs	iron-response elements
IRF1	interferon regulatory factor 1
IRP1/2	iron regulatory proteins 1/2
JAK	tyrosine-protein kinase JAK
JNK	C-Jun N-terminal kinase
MAVS	adaptor protein mitochondrial antiviral signaling
MCM2/7	minichromosome maintenance helicase complex
MDA-5	melanoma differentiation Ag-5
MDC-1	mediator of DNA damage checkpoint protein 1
MDR1	multidrug resistance 1
MHC	major histocompatibility complex
MRE11	meiotic recombination 11 homolog 1
MRN	MRE11–RAD50–NBS1 complex
mtDNA	mitochondrial DNA
NBS1	Nibrin
nDNA	nuclear DNA
NFAT	nuclear factor of activated T-lymphocytes
NF-κB	nuclear factor kappa-light-chain-enhancer of activated B cells
NHEJ	non-homologous end joining
NK	natural killer cell
NLRP3	NACHT, LRR, and PYD domains-containing protein 3
NOXA	phorbol-12-myristate-13-acetate-induced protein 1
NRF2	nuclear factor erythroid 2-related factor 2
P2RX7	P2X purinoceptor
PARP-1	poly(ADP-ribose) polymerase 1
PARs	poly(ADP-ribose) polymers
PD-L1	programmed death ligand 1
PEG	polyethylene glycol
PFNs	Perforins
PG-P	P-glycoprotein
PIKKs	phosphoinositide 3-kinase-related protein kinases
PNKP	polynucleotide kinase phosphatase
pRB	retinoblastoma protein
PRDX2	peroxiredoxin-2
PUMA	isoform 1 of Bcl-2-binding component 3, isoforms 1/2
RHINO	RAD9, RAD1, HUS1 interacting nuclear orphan
RIG-1	retinoic acid inducible gene-I
RNS	reactive nitrogen species
ROS	reactive oxygen species
RPA	replication protein A
SASP	senescence-associated secretory phenotype
SOD2	superoxide dismutase 2
SMAC	secondary mitochondria-derived activator of caspases
SSBR	single-strand break repair
SSBs	single-strand breaks
ssDNA	single-stranded DNA
STAT1/2/3	signal transducer and activator of transcription 1/2/3
TAMs	tumor-associated macrophages
TPD-1	tyrosyl-DNA phosphodiesterase 1
TIS	therapy-induced senescence
TLR4	Toll-like receptor 4
TNFR1	tumor necrosis factor receptor 1
TNF-α	tumor necrosis factor α
TOPB1	topoisomerase 2-binding protein 1
TP53	cellular tumor antigen p53
TRAIL	TNF-related apoptosis inducing ligand
TRX1	thioredoxin-1
XRCC1	X-ray repair cross-complementing group-1

## References

[1] Mohseni M., Samadi N., Ghanbari P., Yousefi B., Tabasinezhad M., Sharifi S., Nazemiyeh H. (2016). Co-Treatment by Docetaxel and Vinblastine Breaks down P-Glycoprotein Mediated Chemo-Resistance. Iran. J. Basic Med. Sci..

[2] Dallavalle S., Dobričić V., Lazzarato L., Gazzano E., Machuqueiro M., Pajeva I., Tsakovska I., Zidar N., Fruttero R. (2020). Improvement of Conventional Anti-Cancer Drugs as New Tools against Multidrug Resistant Tumors. Drug Resist. Updat..

[3] Bukowski K., Kciuk M., Kontek R. (2020). Mechanisms of Multidrug Resistance in Cancer Chemotherapy. Int. J. Mol. Sci..

[4] Carvalho C., Santos R.X., Cardoso S., Correia S., Oliveira P.J., Santos M.S., Moreira P.I. (2009). Doxorubicin: The Good, the Bad and the Ugly Effect. Curr. Med. Chem..

[5] Rawat P.S., Jaiswal A., Khurana A., Bhatti J.S., Navik U. (2021). Doxorubicin-Induced Cardiotoxicity: An Update on the Molecular Mechanism and Novel Therapeutic Strategies for Effective Management. Biomed. Pharmacother..

[6] Thorn C.F., Oshiro C., Marsh S., Hernandez-Boussard T., McLeod H., Klein T.E., Altman R.B. (2011). Doxorubicin Pathways: Pharmacodynamics and Adverse Effects. Pharm. Genom..

[7] Shi Y., Moon M., Dawood S., McManus B., Liu P.P. (2011). Mechanisms and Management of Doxorubicin Cardiotoxicity. Herz.

[8] Wenningmann N., Knapp M., Ande A., Vaidya T.R., Ait-Oudhia S. (2019). Insights into Doxorubicin-Induced Cardiotoxicity: Molecular Mechanisms, Preventive Strategies, and Early Monitoring. Mol. Pharmacol..

[9] Sheibani M., Azizi Y., Shayan M., Nezamoleslami S., Eslami F., Farjoo M.H., Dehpour A.R. (2022). Doxorubicin-Induced Cardiotoxicity: An Overview on Pre-Clinical Therapeutic Approaches. Cardiovasc. Toxicol..

[10] Kalyanaraman B. (2020). Teaching the Basics of the Mechanism of Doxorubicin-Induced Cardiotoxicity: Have We Been Barking up the Wrong Tree?. Redox Biol..

[11] Pugazhendhi A., Edison T.N.J.I., Velmurugan B.K., Jacob J.A., Karuppusamy I. (2018). Toxicity of Doxorubicin (Dox) to Different Experimental Organ Systems. Life Sci..

[12] Tacar O., Sriamornsak P., Dass C.R. (2013). Doxorubicin: An Update on Anticancer Molecular Action, Toxicity and Novel Drug Delivery Systems. J. Pharm. Pharmacol..

[13] Mohan U.P., Pichiah P.B.T., Iqbal S.T.A., Arunachalam S. (2021). Mechanisms of Doxorubicin-Mediated Reproductive Toxicity—A Review. Reprod. Toxicol..

[14] Alhowail A.H., Bloemer J., Majrashi M., Pinky P.D., Bhattacharya S., Yongli Z., Bhattacharya D., Eggert M., Woodie L., Buabeid M.A. (2019). Doxorubicin-Induced Neurotoxicity Is Associated with Acute Alterations in Synaptic Plasticity, Apoptosis, and Lipid Peroxidation. Toxicol. Mech. Methods.

[15] Tangpong J., Miriyala S., Noel T., Sinthupibulyakit C., Jungsuwadee P., St Clair D.K. (2011). Doxorubicin-Induced Central Nervous System Toxicity and Protection by Xanthone Derivative of Garcinia Mangostana. Neuroscience.

[16] Prasanna P.L., Renu K., Valsala Gopalakrishnan A. (2020). New Molecular and Biochemical Insights of Doxorubicin-Induced Hepatotoxicity. Life Sci..

[17] Ayla S., Seckin I., Tanriverdi G., Cengiz M., Eser M., Soner B.C., Oktem G. (2011). Doxorubicin Induced Nephrotoxicity: Protective Effect of Nicotinamide. Int. J. Cell Biol..

[18] Manil L., Couvreur P., Mahieu P. (1995). Acute Renal Toxicity of Doxorubicin (Adriamycin)-Loaded Cyanoacrylate Nanoparticles. Pharm. Res..

[19] Lahoti T.S., Patel D., Thekkemadom V., Beckett R., Ray S.D. (2012). Doxorubicin-Induced in Vivo Nephrotoxicity Involves Oxidative Stress-Mediated Multiple pro- and Anti-Apoptotic Signaling Pathways. Curr. Neurovasc. Res..

[20] van der Zanden S.Y., Qiao X., Neefjes J. (2021). New Insights into the Activities and Toxicities of the Old Anticancer Drug Doxorubicin. FEBS J..

[21] Varela-López A., Battino M., Navarro-Hortal M.D., Giampieri F., Forbes-Hernández T.Y., Romero-Márquez J.M., Collado R., Quiles J.L. (2019). An Update on the Mechanisms Related to Cell Death and Toxicity of Doxorubicin and the Protective Role of Nutrients. Food Chem. Toxicol..

[22] Moustafa I., Viljoen M., Perumal-Pillay V.A., Oosthuizen F. (2022). Critical Appraisal of Clinical Guidelines for Prevention and Management of Doxorubicin-Induced Cardiotoxicity. J. Oncol. Pharm. Pract..

[23] Benzer F., Kandemir F.M., Kucukler S., Comaklı S., Caglayan C. (2018). Chemoprotective Effects of Curcumin on Doxorubicin-Induced Nephrotoxicity in Wistar Rats: By Modulating Inflammatory Cytokines, Apoptosis, Oxidative Stress and Oxidative DNA Damage. Arch. Physiol. Biochem..

[24] Rashid S., Ali N., Nafees S., Ahmad S.T., Arjumand W., Hasan S.K., Sultana S. (2013). Alleviation of Doxorubicin-Induced Nephrotoxicity and Hepatotoxicity by Chrysin in Wistar Rats. Toxicol. Mech. Methods.

[25] Ibrahim Fouad G., Ahmed K.A. (2021). Neuroprotective Potential of Berberine Against Doxorubicin-Induced Toxicity in Rat’s Brain. Neurochem. Res..

[26] Shafei A., El-Bakly W., Sobhy A., Wagdy O., Reda A., Aboelenin O., Marzouk A., El Habak K., Mostafa R., Ali M.A. (2017). A Review on the Efficacy and Toxicity of Different Doxorubicin Nanoparticles for Targeted Therapy in Metastatic Breast Cancer. Biomed. Pharmacother..

[27] Prados J., Melguizo C., Ortiz R., Vélez C., Alvarez P.J., Arias J.L., Ruíz M.A., Gallardo V., Aranega A. (2012). Doxorubicin-Loaded Nanoparticles: New Advances in Breast Cancer Therapy. Anticancer Agents Med. Chem..

[28] Zhao N., Woodle M.C., Mixson A.J. (2018). Advances in Delivery Systems for Doxorubicin. J. Nanomed. Nanotechnol..

[29] Kanwal U., Irfan Bukhari N., Ovais M., Abass N., Hussain K., Raza A. (2018). Advances in Nano-Delivery Systems for Doxorubicin: An Updated Insight. J. Drug Target..

[30] Ansari L., Shiehzadeh F., Taherzadeh Z., Nikoofal-Sahlabadi S., Momtazi-Borojeni A.A., Sahebkar A., Eslami S. (2017). The Most Prevalent Side Effects of Pegylated Liposomal Doxorubicin Monotherapy in Women with Metastatic Breast Cancer: A Systematic Review of Clinical Trials. Cancer Gene Ther..

[31] Miguel R.D.A., Hirata A.S., Jimenez P.C., Lopes L.B., Costa-Lotufo L.V. (2022). Beyond Formulation: Contributions of Nanotechnology for Translation of Anticancer Natural Products into New Drugs. Pharmaceutics.

[32] Mukhtar M., Bilal M., Rahdar A., Barani M., Arshad R., Behl T., Brisc C., Banica F., Bungau S. (2020). Nanomaterials for Diagnosis and Treatment of Brain Cancer: Recent Updates. Chemosensors.

[33] Skarbek C., Serra S., Maslah H., Rascol E., Labruère R. (2019). Arylboronate Prodrugs of Doxorubicin as Promising Chemotherapy for Pancreatic Cancer. Bioorg. Chem..

[34] Zhong Y.-J., Shao L.-H., Li Y. (2013). Cathepsin B-Cleavable Doxorubicin Prodrugs for Targeted Cancer Therapy (Review). Int. J. Oncol..

[35] Garsky V.M., Lumma P.K., Feng D.M., Wai J., Ramjit H.G., Sardana M.K., Oliff A., Jones R.E., DeFeo-Jones D., Freidinger R.M. (2001). The Synthesis of a Prodrug of Doxorubicin Designed to Provide Reduced Systemic Toxicity and Greater Target Efficacy. J. Med. Chem..

[36] DeFeo-Jones D., Garsky V.M., Wong B.K., Feng D.M., Bolyar T., Haskell K., Kiefer D.M., Leander K., McAvoy E., Lumma P. (2000). A Peptide-Doxorubicin “prodrug” Activated by Prostate-Specific Antigen Selectively Kills Prostate Tumor Cells Positive for Prostate-Specific Antigen in Vivo. Nat. Med..

[37] Albright C.F., Graciani N., Han W., Yue E., Stein R., Lai Z., Diamond M., Dowling R., Grimminger L., Zhang S.-Y. (2005). Matrix Metalloproteinase-Activated Doxorubicin Prodrugs Inhibit HT1080 Xenograft Growth Better than Doxorubicin with Less Toxicity. Mol. Cancer Ther..

[38] Bao Y., Yin M., Hu X., Zhuang X., Sun Y., Guo Y., Tan S., Zhang Z. (2016). A Safe, Simple and Efficient Doxorubicin Prodrug Hybrid Micelle for Overcoming Tumor Multidrug Resistance and Targeting Delivery. J. Control. Release.

[39] Farquhar D., Newman R.A., Zuckerman J.E., Andersson B.S. (1991). Doxorubicin Analogues Incorporating Chemically Reactive Substituents. J. Med. Chem..

[40] Weiss R.B. (1992). The Anthracyclines: Will We Ever Find a Better Doxorubicin?. Semin. Oncol..

[41] Brown J.R., Imam S.H. (1984). Recent Studies on Doxorubicin and Its Analogues. Prog. Med. Chem..

[42] Giuliani F.C., Kaplan N.O. (1980). New Doxorubicin Analogs Active against Doxorubicin-Resistant Colon Tumor Xenografts in the Nude Mouse. Cancer Res..

[43] Lang D.K., Kaur R., Arora R., Saini B., Arora S. (2020). Nitrogen-Containing Heterocycles as Anticancer Agents: An Overview. Anticancer Agents Med. Chem..

[44] Sohail M., Sun Z., Li Y., Gu X., Xu H. (2021). Research Progress in Strategies to Improve the Efficacy and Safety of Doxorubicin for Cancer Chemotherapy. Expert Rev. Anticancer Ther..

[45] Zujewski J.A., Eng-Wong J., O’Shaughnessy J., Venzon D., Chow C., Danforth D., Kohler D.R., Cusack G., Riseberg D., Cowan K.H. (2003). A Pilot Study of Dose Intense Doxorubicin and Cyclophosphamide Followed by Infusional Paclitaxel in High-Risk Primary Breast Cancer. Breast Cancer Res. Treat..

[46] Liu X., Jiang S., Wang H., Wu X., Yan W., Chen Y., Xu Y., Wang C., Yao W., Wang J. (2022). Pegylated Liposomal Doxorubicin Combined with Ifosfamide for Treating Advanced or Metastatic Soft-Tissue Sarcoma: A Prospective, Single-Arm Phase II Study. Clin. Cancer Res..

[47] Pautier P., Italiano A., Piperno-Neumann S., Chevreau C., Penel N., Firmin N., Boudou-Rouquette P., Bertucci F., Balleyguier C., Lebrun-Ly V. (2022). Doxorubicin Alone versus Doxorubicin with Trabectedin Followed by Trabectedin Alone as First-Line Therapy for Metastatic or Unresectable Leiomyosarcoma (LMS-04): A Randomised, Multicentre, Open-Label Phase 3 Trial. Lancet Oncol..

[48] Picardi M., Giordano C., Pugliese N., Esposito M., Fatigati M., Muriano F., Rascato M.G., Pepa R.D., D’Ambrosio A., Vigliar E. (2022). Liposomal Doxorubicin Supercharge-Containing Front-Line Treatment in Patients with Advanced-Stage Diffuse Large B-Cell Lymphoma or Classical Hodgkin Lymphoma: Preliminary Results of a Single-Centre Phase II Study. Br. J. Haematol..

[49] Prakash S.S., Nagarkar R.V., Puligundla K.C., Lokesh K.N., Boya R.R., Patel A.B., Goyal L., Thoke A., Patel J.G., Mehta A.O. (2022). Bioequivalence of a Hybrid Pegylated Liposomal Doxorubicin Hydrochloride Injection and Caelyx^®^: A Single-Dose, Randomized, Multicenter, Open-Label, Two-Period Crossover Study in Patients with Advanced Ovarian Cancer. Eur. J. Pharm. Sci..

[50] Wang T., Tang J., Yang H., Yin R., Zhang J., Zhou Q., Liu Z., Cao L., Li L., Huang Y. (2022). Effect of Apatinib Plus Pegylated Liposomal Doxorubicin vs Pegylated Liposomal Doxorubicin Alone on Platinum-Resistant Recurrent Ovarian Cancer: The APPROVE Randomized Clinical Trial. JAMA Oncol..

[51] Lee Y.J., Seol A., Lee M., Kim J.-W., Kim H.S., Kim K., Suh D.H., Kim S., Kim S.W., Lee J.-Y. (2022). A Phase II Trial to Evaluate the Efficacy of Bortezomib and Liposomal Doxorubicin in Patients With BRCA Wild-Type Platinum-Resistant Recurrent Ovarian Cancer (KGOG 3044/EBLIN). In Vivo.

[52] Elfadadny A., Ragab R.F., Hamada R., Al Jaouni S.K., Fu J., Mousa S.A., El-Far A.H. (2023). Natural Bioactive Compounds-Doxorubicin Combinations Targeting Topoisomerase II-Alpha: Anticancer Efficacy and Safety. Toxicol. Appl. Pharmacol..

[53] Qiu C., Wu Y., Shi Q., Guo Q., Zhang J., Meng Y., Wang C., Xia F., Wang J., Xu C. (2023). Advanced Strategies for Nucleic Acids and Small-Molecular Drugs in Combined Anticancer Therapy. Int. J. Biol. Sci..

[54] de Padua T.C., Marandino L., Raggi D., Hallanger-Johnson J., Kutikov A., Spiess P.E., Necchi A. (2023). A Systematic Review of Published Clinical Trials in the Systemic Treatment of Adrenocortical Carcinoma: An Initiative Led on Behalf of the Global Society of Rare Genitourinary Tumors. Clin. Genitourin. Cancer.

[55] Chen Y., Xu H., Shan N., Qu H. (2022). Pegylated Liposomal Doxorubicin (PLD)-Containing Regimen as a Novel Treatment of Monomorphic Epithelial Intestinal T-Cell Lymphoma (MEITL): A Case Report and Review of Literature. Medicine.

[56] Younes M., Mardirossian R., Rizk L., Fazlian T., Khairallah J.P., Sleiman C., Naim H.Y., Rizk S. (2022). The Synergistic Effects of Curcumin and Chemotherapeutic Drugs in Inhibiting Metastatic, Invasive and Proliferative Pathways. Plants.

[57] Kciuk M., Kołat D., Kałuzińska-Kołat Ż., Gawrysiak M., Drozda R., Celik I., Kontek R. (2023). PD-1/PD-L1 and DNA Damage Response in Cancer. Cells.

[58] Howerton S.B., Nagpal A., Williams L.D. (2003). Surprising Roles of Electrostatic Interactions in DNA-Ligand Complexes. Biopolymers.

[59] Lipscomb L.A., Peek M.E., Zhou F.X., Bertrand J.A., VanDerveer D., Williams L.D. (1994). Water Ring Structure at DNA Interfaces: Hydration and Dynamics of DNA-Anthracycline Complexes. Biochemistry.

[60] Zlatanova J., Victor J.-M. (2009). How Are Nucleosomes Disrupted during Transcription Elongation?. HFSP J..

[61] Yang F., Kemp C.J., Henikoff S. (2013). Doxorubicin Enhances Nucleosome Turnover around Promoters. Curr. Biol..

[62] Salerno D., Brogioli D., Cassina V., Turchi D., Beretta G.L., Seruggia D., Ziano R., Zunino F., Mantegazza F. (2010). Magnetic Tweezers Measurements of the Nanomechanical Properties of DNA in the Presence of Drugs. Nucleic Acids Res..

[63] Yang F., Teves S.S., Kemp C.J., Henikoff S. (2014). Doxorubicin, DNA Torsion, and Chromatin Dynamics. Biochim. Biophys. Acta.

[64] Swift L.P., Rephaeli A., Nudelman A., Phillips D.R., Cutts S.M. (2006). Doxorubicin-DNA Adducts Induce a Non-Topoisomerase II-Mediated Form of Cell Death. Cancer Res..

[65] Forrest R.A., Swift L.P., Rephaeli A., Nudelman A., Kimura K.-I., Phillips D.R., Cutts S.M. (2012). Activation of DNA Damage Response Pathways as a Consequence of Anthracycline-DNA Adduct Formation. Biochem. Pharmacol..

[66] Coldwell K.E., Cutts S.M., Ognibene T.J., Henderson P.T., Phillips D.R. (2008). Detection of Adriamycin-DNA Adducts by Accelerator Mass Spectrometry at Clinically Relevant Adriamycin Concentrations. Nucleic Acids Res..

[67] Kciuk M., Marciniak B., Kontek R. (2020). Irinotecan—Still an Important Player in Cancer Chemotherapy: A Comprehensive Overview. Int. J. Mol. Sci..

[68] Pommier Y., Leo E., Zhang H., Marchand C. (2010). DNA Topoisomerases and Their Poisoning by Anticancer and Antibacterial Drugs. Chem. Biol..

[69] Ross W.E., Glaubiger D.L., Kohn K.W. (1978). Protein-Associated DNA Breaks in Cells Treated with Adriamycin or Ellipticine. Biochim. Biophys. Acta.

[70] Tewey K.M., Rowe T.C., Yang L., Halligan B.D., Liu L.F. (1984). Adriamycin-Induced DNA Damage Mediated by Mammalian DNA Topoisomerase II. Science.

[71] Fokas E., Prevo R., Hammond E.M., Brunner T.B., McKenna W.G., Muschel R.J. (2014). Targeting ATR in DNA Damage Response and Cancer Therapeutics. Cancer Treat. Rev..

[72] Lecona E., Fernandez-Capetillo O. (2018). Targeting ATR in Cancer. Nat. Rev. Cancer.

[73] Maréchal A., Zou L. (2013). DNA Damage Sensing by the ATM and ATR Kinases. Cold Spring Harb. Perspect. Biol..

[74] Saldivar J.C., Cortez D., Cimprich K.A. (2017). The Essential Kinase ATR: Ensuring Faithful Duplication of a Challenging Genome. Nat. Rev. Mol. Cell Biol..

[75] Ma A., Dai X. (2018). The Relationship between DNA Single-Stranded Damage Response and Double-Stranded Damage Response. Cell Cycle.

[76] Kciuk M., Gielecińska A., Kołat D., Kałuzińska Ż., Kontek R. (2022). Cancer-Associated Transcription Factors in DNA Damage Response. Biochim. Biophys. Acta Rev. Cancer.

[77] Kciuk M., Gielecińska A., Mujwar S., Mojzych M., Kontek R. (2022). Cyclin-Dependent Kinases in DNA Damage Response. Biochim. Biophys. Acta Rev. Cancer.

[78] Kciuk M., Marciniak B., Mojzych M., Kontek R. (2020). Focus on UV-Induced DNA Damage and Repair—Disease Relevance and Protective Strategies. Int. J. Mol. Sci..

[79] Cimprich K.A., Cortez D. (2008). ATR: An Essential Regulator of Genome Integrity. Nat. Rev. Mol. Cell Biol..

[80] Mei C., Lei L., Tan L.-M., Xu X.-J., He B.-M., Luo C., Yin J.-Y., Li X., Zhang W., Zhou H.-H. (2020). The Role of Single Strand Break Repair Pathways in Cellular Responses to Camptothecin Induced DNA Damage. Biomed. Pharmacother..

[81] Abbotts R., Wilson D.M. (2017). Coordination of DNA Single Strand Break Repair. Free Radic. Biol. Med..

[82] Fortini P., Dogliotti E. (2007). Base Damage and Single-Strand Break Repair: Mechanisms and Functional Significance of Short- and Long-Patch Repair Subpathways. DNA Repair..

[83] Caldecott K.W. (2007). Mammalian Single-Strand Break Repair: Mechanisms and Links with Chromatin. DNA Repair..

[84] Spiegel J.O., Van Houten B., Durrant J.D. (2021). PARP1: Structural Insights and Pharmacological Targets for Inhibition. DNA Repair..

[85] van den Bosch M., Bree R.T., Lowndes N.F. (2003). The MRN Complex: Coordinating and Mediating the Response to Broken Chromosomes. EMBO Rep..

[86] Qiu S., Huang J. (2021). MRN Complex Is an Essential Effector of DNA Damage Repair. J. Zhejiang Univ. Sci. B.

[87] Lamarche B.J., Orazio N.I., Weitzman M.D. (2010). The MRN Complex in Double-Strand Break Repair and Telomere Maintenance. FEBS Lett..

[88] Yue X., Bai C., Xie D., Ma T., Zhou P.-K. (2020). DNA-PKcs: A Multi-Faceted Player in DNA Damage Response. Front. Genet..

[89] Dylgjeri E., Knudsen K.E. (2022). DNA-PKcs: A Targetable Protumorigenic Protein Kinase. Cancer Res..

[90] Amani J., Gorjizadeh N., Younesi S., Najafi M., Ashrafi A.M., Irian S., Gorjizadeh N., Azizian K. (2021). Cyclin-Dependent Kinase Inhibitors (CDKIs) and the DNA Damage Response: The Link between Signaling Pathways and Cancer. DNA Repair..

[91] Kciuk M., Gielecińska A., Mujwar S., Mojzych M., Kontek R. (2022). Cyclin-Dependent Kinase Synthetic Lethality Partners in DNA Damage Response. Int. J. Mol. Sci..

[92] Lim S., Kaldis P. (2013). Cdks, Cyclins and CKIs: Roles beyond Cell Cycle Regulation. Development.

[93] Kurz E.U., Douglas P., Lees-Miller S.P. (2004). Doxorubicin Activates ATM-Dependent Phosphorylation of Multiple Downstream Targets in Part through the Generation of Reactive Oxygen Species. J. Biol. Chem..

[94] Théard D., Coisy M., Ducommun B., Concannon P., Darbon J.M. (2001). Etoposide and Adriamycin but Not Genistein Can Activate the Checkpoint Kinase Chk2 Independently of ATM/ATR. Biochem. Biophys. Res. Commun..

[95] Ghelli Luserna Di Rorà A., Ghetti M., Ledda L., Ferrari A., Bocconcelli M., Padella A., Napolitano R., Fontana M.C., Liverani C., Imbrogno E. Exploring the ATR-CHK1 Pathway in the Response of Doxorubicin-Induced DNA Damages in Acute Lymphoblastic Leukemia Cells. Cell Biol. Toxicol..

[96] Batey M.A., Zhao Y., Kyle S., Richardson C., Slade A., Martin N.M.B., Lau A., Newell D.R., Curtin N.J. (2013). Preclinical Evaluation of a Novel ATM Inhibitor, KU59403, in Vitro and in Vivo in P53 Functional and Dysfunctional Models of Human Cancer. Mol. Cancer Ther..

[97] Cui J., Dean D., Hornicek F.J., Pollock R.E., Hoffman R.M., Duan Z. (2022). ATR Inhibition Sensitizes Liposarcoma to Doxorubicin by Increasing DNA Damage. Am. J. Cancer Res..

[98] Baranski Z., Booij T.H., Cleton-Jansen A.-M., Price L.S., van de Water B., Bovée J.V.M.G., Hogendoorn P.C.W., Danen E.H.J. (2015). Aven-Mediated Checkpoint Kinase Control Regulates Proliferation and Resistance to Chemotherapy in Conventional Osteosarcoma. J. Pathol..

[99] Chung S.W., Kim G.C., Kweon S., Lee H., Choi J.U., Mahmud F., Chang H.W., Kim J.W., Son W.-C., Kim S.Y. (2018). Metronomic Oral Doxorubicin in Combination of Chk1 Inhibitor MK-8776 for P53-Deficient Breast Cancer Treatment. Biomaterials.

[100] Weng M.-T., Tung T.-H., Lee J.-H., Wei S.-C., Lin H.-L., Huang Y.-J., Wong J.-M., Luo J., Sheu J.-C. (2015). Enhancer of Rudimentary Homolog Regulates DNA Damage Response in Hepatocellular Carcinoma. Sci. Rep..

[101] Davidson D., Grenier J., Martinez-Marignac V., Amrein L., Shawi M., Tokars M., Aloyz R., Panasci L. (2012). Effects of the Novel DNA Dependent Protein Kinase Inhibitor, IC486241, on the DNA Damage Response to Doxorubicin and Cisplatin in Breast Cancer Cells. Investig. New Drugs.

[102] Park H.J., Bae J.S., Kim K.M., Moon Y.J., Park S.-H., Ha S.H., Hussein U.K., Zhang Z., Park H.S., Park B.-H. (2018). The PARP Inhibitor Olaparib Potentiates the Effect of the DNA Damaging Agent Doxorubicin in Osteosarcoma. J. Exp. Clin. Cancer Res..

[103] Kim H.-S., Lee Y.-S., Kim D.-K. (2009). Doxorubicin Exerts Cytotoxic Effects through Cell Cycle Arrest and Fas-Mediated Cell Death. Pharmacology.

[104] Bar-On O., Shapira M., Hershko D.D. (2007). Differential Effects of Doxorubicin Treatment on Cell Cycle Arrest and Skp2 Expression in Breast Cancer Cells. Anticancer Drugs.

[105] Ling Y.H., el-Naggar A.K., Priebe W., Perez-Soler R. (1996). Cell Cycle-Dependent Cytotoxicity, G2/M Phase Arrest, and Disruption of P34cdc2/Cyclin B1 Activity Induced by Doxorubicin in Synchronized P388 Cells. Mol. Pharmacol..

[106] Lüpertz R., Wätjen W., Kahl R., Chovolou Y. (2010). Dose- and Time-Dependent Effects of Doxorubicin on Cytotoxicity, Cell Cycle and Apoptotic Cell Death in Human Colon Cancer Cells. Toxicology.

[107] Sarniak A., Lipińska J., Tytman K., Lipińska S. (2016). Endogenous Mechanisms of Reactive Oxygen Species (ROS) Generation. Postep. Hig. Med. Dosw. (Online).

[108] Zorov D.B., Juhaszova M., Sollott S.J. (2014). Mitochondrial Reactive Oxygen Species (ROS) and ROS-Induced ROS Release. Physiol. Rev..

[109] Cadet J., Douki T., Ravanat J.-L. (2010). Oxidatively Generated Base Damage to Cellular DNA. Free Radic. Biol. Med..

[110] Cadet J., Davies K.J.A. (2017). Oxidative DNA Damage & Repair: An Introduction. Free Radic. Biol. Med..

[111] Simon H.U., Haj-Yehia A., Levi-Schaffer F. (2000). Role of Reactive Oxygen Species (ROS) in Apoptosis Induction. Apoptosis.

[112] Redza-Dutordoir M., Averill-Bates D.A. (2016). Activation of Apoptosis Signalling Pathways by Reactive Oxygen Species. Biochim. Biophys. Acta.

[113] Kuczler M.D., Olseen A.M., Pienta K.J., Amend S.R. (2021). ROS-Induced Cell Cycle Arrest as a Mechanism of Resistance in Polyaneuploid Cancer Cells (PACCs). Prog. Biophys. Mol. Biol..

[114] Chatterjee S., Kundu S., Sengupta S., Bhattacharyya A. (2009). Divergence to Apoptosis from ROS Induced Cell Cycle Arrest: Effect of Cadmium. Mutat. Res..

[115] Victorelli S., Passos J.F. (2019). Reactive Oxygen Species Detection in Senescent Cells. Methods Mol. Biol..

[116] Benkafadar N., François F., Affortit C., Casas F., Ceccato J.-C., Menardo J., Venail F., Malfroy-Camine B., Puel J.-L., Wang J. (2019). ROS-Induced Activation of DNA Damage Responses Drives Senescence-Like State in Postmitotic Cochlear Cells: Implication for Hearing Preservation. Mol. Neurobiol..

[117] Davalli P., Mitic T., Caporali A., Lauriola A., D’Arca D. (2016). ROS, Cell Senescence, and Novel Molecular Mechanisms in Aging and Age-Related Diseases. Oxidative Med. Cell. Longev..

[118] Bertram C., Hass R. (2008). Cellular Responses to Reactive Oxygen Species-Induced DNA Damage and Aging. Biol. Chem..

[119] Gilliam L.A.A., Moylan J.S., Patterson E.W., Smith J.D., Wilson A.S., Rabbani Z., Reid M.B. (2012). Doxorubicin Acts via Mitochondrial ROS to Stimulate Catabolism in C2C12 Myotubes. Am. J. Physiol. Cell Physiol..

[120] Goormaghtigh E., Huart P., Praet M., Brasseur R., Ruysschaert J.M. (1990). Structure of the Adriamycin-Cardiolipin Complex. Role in Mitochondrial Toxicity. Biophys. Chem..

[121] Gorini S., De Angelis A., Berrino L., Malara N., Rosano G., Ferraro E. (2018). Chemotherapeutic Drugs and Mitochondrial Dysfunction: Focus on Doxorubicin, Trastuzumab, and Sunitinib. Oxidative Med. Cell. Longev..

[122] Montalvo R.N., Doerr V., Min K., Szeto H.H., Smuder A.J. (2020). Doxorubicin-Induced Oxidative Stress Differentially Regulates Proteolytic Signaling in Cardiac and Skeletal Muscle. Am. J. Physiol. Regul. Integr. Comp. Physiol..

[123] Kalivendi S.V., Konorev E.A., Cunningham S., Vanamala S.K., Kaji E.H., Joseph J., Kalyanaraman B. (2005). Doxorubicin Activates Nuclear Factor of Activated T-Lymphocytes and Fas Ligand Transcription: Role of Mitochondrial Reactive Oxygen Species and Calcium. Biochem. J..

[124] Zhao L., Zhang B. (2017). Doxorubicin Induces Cardiotoxicity through Upregulation of Death Receptors Mediated Apoptosis in Cardiomyocytes. Sci. Rep..

[125] McSweeney K.M., Bozza W.P., Alterovitz W.-L., Zhang B. (2019). Transcriptomic Profiling Reveals P53 as a Key Regulator of Doxorubicin-Induced Cardiotoxicity. Cell Death Discov..

[126] Zhou S., Palmeira C.M., Wallace K.B. (2001). Doxorubicin-Induced Persistent Oxidative Stress to Cardiac Myocytes. Toxicol. Lett..

[127] Zhang S., Liu X., Bawa-Khalfe T., Lu L.-S., Lyu Y.L., Liu L.F., Yeh E.T.H. (2012). Identification of the Molecular Basis of Doxorubicin-Induced Cardiotoxicity. Nat. Med..

[128] Wang Z., Wang J., Xie R., Liu R., Lu Y. (2015). Mitochondria-Derived Reactive Oxygen Species Play an Important Role in Doxorubicin-Induced Platelet Apoptosis. Int. J. Mol. Sci..

[129] Kim S.-Y., Kim S.-J., Kim B.-J., Rah S.-Y., Chung S.M., Im M.-J., Kim U.-H. (2006). Doxorubicin-Induced Reactive Oxygen Species Generation and Intracellular Ca2+ Increase Are Reciprocally Modulated in Rat Cardiomyocytes. Exp. Mol. Med..

[130] Ma J., Wang Y., Zheng D., Wei M., Xu H., Peng T. (2013). Rac1 Signalling Mediates Doxorubicin-Induced Cardiotoxicity through Both Reactive Oxygen Species-Dependent and -Independent Pathways. Cardiovasc. Res..

[131] Asensio-López M.C., Soler F., Pascual-Figal D., Fernández-Belda F., Lax A. (2017). Doxorubicin-Induced Oxidative Stress: The Protective Effect of Nicorandil on HL-1 Cardiomyocytes. PLoS ONE.

[132] Antonucci S., Di Sante M., Tonolo F., Pontarollo L., Scalcon V., Alanova P., Menabò R., Carpi A., Bindoli A., Rigobello M.P. (2021). The Determining Role of Mitochondrial Reactive Oxygen Species Generation and Monoamine Oxidase Activity in Doxorubicin-Induced Cardiotoxicity. Antioxid. Redox Signal..

[133] Songbo M., Lang H., Xinyong C., Bin X., Ping Z., Liang S. (2019). Oxidative Stress Injury in Doxorubicin-Induced Cardiotoxicity. Toxicol. Lett..

[134] Olson R.D., Mushlin P.S. (1990). Doxorubicin Cardiotoxicity: Analysis of Prevailing Hypotheses. FASEB J..

[135] Dos Santos Arruda F., Tomé F.D., Miguel M.P., de Menezes L.B., Nagib P.R.A., Campos E.C., Soave D.F., Celes M.R.N. (2019). Doxorubicin-Induced Cardiotoxicity and Cardioprotective Agents: Classic and New Players in the Game. Curr. Pharm. Des..

[136] Cappetta D., De Angelis A., Sapio L., Prezioso L., Illiano M., Quaini F., Rossi F., Berrino L., Naviglio S., Urbanek K. (2017). Oxidative Stress and Cellular Response to Doxorubicin: A Common Factor in the Complex Milieu of Anthracycline Cardiotoxicity. Oxidative Med. Cell. Longev..

[137] Guo Z., Kozlov S., Lavin M.F., Person M.D., Paull T.T. (2010). ATM Activation by Oxidative Stress. Science.

[138] Xie X., Zhang Y., Wang Z., Wang S., Jiang X., Cui H., Zhou T., He Z., Feng H., Guo Q. (2021). ATM at the Crossroads of Reactive Oxygen Species and Autophagy. Int. J. Biol. Sci..

[139] Sarkar A., Gandhi V. (2021). Activation of ATM Kinase by ROS Generated during Ionophore-Induced Mitophagy in Human T and B Cell Malignancies. Mol. Cell. Biochem..

[140] Tsang W.P., Chau S.P.Y., Kong S.K., Fung K.P., Kwok T.T. (2003). Reactive Oxygen Species Mediate Doxorubicin Induced P53-Independent Apoptosis. Life Sci..

[141] Pilco-Ferreto N., Calaf G.M. (2016). Influence of Doxorubicin on Apoptosis and Oxidative Stress in Breast Cancer Cell Lines. Int. J. Oncol..

[142] Navarro R., Busnadiego I., Ruiz-Larrea M.B., Ruiz-Sanz J.I. (2006). Superoxide Anions Are Involved in Doxorubicin-Induced ERK Activation in Hepatocyte Cultures. Ann. N. Y. Acad. Sci..

[143] Filippova M., Filippov V., Williams V.M., Zhang K., Kokoza A., Bashkirova S., Duerksen-Hughes P. (2014). Cellular Levels of Oxidative Stress Affect the Response of Cervical Cancer Cells to Chemotherapeutic Agents. Biomed. Res. Int..

[144] Luanpitpong S., Chanvorachote P., Nimmannit U., Leonard S.S., Stehlik C., Wang L., Rojanasakul Y. (2012). Mitochondrial Superoxide Mediates Doxorubicin-Induced Keratinocyte Apoptosis through Oxidative Modification of ERK and Bcl-2 Ubiquitination. Biochem. Pharmacol..

[145] Aries A., Paradis P., Lefebvre C., Schwartz R.J., Nemer M. (2004). Essential Role of GATA-4 in Cell Survival and Drug-Induced Cardiotoxicity. Proc. Natl. Acad. Sci. USA.

[146] Doroshow J.H. (2019). Mechanisms of Anthracycline-Enhanced Reactive Oxygen Metabolism in Tumor Cells. Oxidative Med. Cell. Longev..

[147] Voulgaridou G.-P., Anestopoulos I., Franco R., Panayiotidis M.I., Pappa A. (2011). DNA Damage Induced by Endogenous Aldehydes: Current State of Knowledge. Mutat. Res..

[148] Antonowicz S., Bodai Z., Wiggins T., Markar S.R., Boshier P.R., Goh Y.M., Adam M.E., Lu H., Kudo H., Rosini F. (2021). Endogenous Aldehyde Accumulation Generates Genotoxicity and Exhaled Biomarkers in Esophageal Adenocarcinoma. Nat. Commun..

[149] Nakamura J., Nakamura M. (2020). DNA-Protein Crosslink Formation by Endogenous Aldehydes and AP Sites. DNA Repair..

[150] Hlaváčová M., Gumulec J., Stračina T., Fojtů M., Raudenská M., Masařík M., Nováková M., Paulová H. (2015). Different Doxorubicin Formulations Affect Plasma 4-Hydroxy-2-Nonenal and Gene Expression of Aldehyde Dehydrogenase 3A1 and Thioredoxin Reductase 2 in Rat. Physiol. Res..

[151] Luo X., Evrovsky Y., Cole D., Trines J., Benson L.N., Lehotay D.C. (1997). Doxorubicin-Induced Acute Changes in Cytotoxic Aldehydes, Antioxidant Status and Cardiac Function in the Rat. Biochim. Biophys. Acta.

[152] Hrelia S., Fiorentini D., Maraldi T., Angeloni C., Bordoni A., Biagi P.L., Hakim G. (2002). Doxorubicin Induces Early Lipid Peroxidation Associated with Changes in Glucose Transport in Cultured Cardiomyocytes. Biochim. Biophys. Acta.

[153] Minotti G., Menna P., Salvatorelli E., Cairo G., Gianni L. (2004). Anthracyclines: Molecular Advances and Pharmacologic Developments in Antitumor Activity and Cardiotoxicity. Pharmacol. Rev..

[154] Senchenkov A., Litvak D.A., Cabot M.C. (2001). Targeting Ceramide Metabolism--a Strategy for Overcoming Drug Resistance. J. Natl. Cancer Inst..

[155] Kawase M., Watanabe M., Kondo T., Yabu T., Taguchi Y., Umehara H., Uchiyama T., Mizuno K., Okazaki T. (2002). Increase of Ceramide in Adriamycin-Induced HL-60 Cell Apoptosis: Detection by a Novel Anti-Ceramide Antibody. Biochim. Biophys. Acta.

[156] Doroshow J.H., Synold T.W., Somlo G., Akman S.A., Gajewski E. (2001). Oxidative DNA Base Modifications in Peripheral Blood Mononuclear Cells of Patients Treated with High-Dose Infusional Doxorubicin. Blood.

[157] Gajewski E., Gaur S., Akman S.A., Matsumoto L., van Balgooy J.N.A., Doroshow J.H. (2007). Oxidative DNA Base Damage in MCF-10A Breast Epithelial Cells at Clinically Achievable Concentrations of Doxorubicin. Biochem. Pharmacol..

[158] García-Ruiz C., Colell A., Marí M., Morales A., Fernández-Checa J.C. (1997). Direct Effect of Ceramide on the Mitochondrial Electron Transport Chain Leads to Generation of Reactive Oxygen Species. Role of Mitochondrial Glutathione. J. Biol. Chem..

[159] Chang Y.C., Fong Y., Tsai E.-M., Chang Y.-G., Chou H.L., Wu C.-Y., Teng Y.-N., Liu T.-C., Yuan S.-S., Chiu C.-C. (2018). Exogenous C8-Ceramide Induces Apoptosis by Overproduction of ROS and the Switch of Superoxide Dismutases SOD1 to SOD2 in Human Lung Cancer Cells. Int. J. Mol. Sci..

[160] Siskind L.J. (2005). Mitochondrial Ceramide and the Induction of Apoptosis. J. Bioenerg. Biomembr..

[161] Woodcock J. (2006). Sphingosine and Ceramide Signalling in Apoptosis. IUBMB Life.

[162] Mullen T.D., Obeid L.M. (2012). Ceramide and Apoptosis: Exploring the Enigmatic Connections between Sphingolipid Metabolism and Programmed Cell Death. Anticancer Agents Med. Chem..

[163] Khodadust R., Alpsoy A., Ünsoy G., GÜndÜz U. (2020). Poly (I:C)- and Doxorubicin-Loaded Magnetic Dendrimeric Nanoparticles Affect the Apoptosis-Related Gene Expressions in MCF-7 Cells. Turk. J. Biol..

[164] Bojko A., Czarnecka-Herok J., Charzynska A., Dabrowski M., Sikora E. (2019). Diversity of the Senescence Phenotype of Cancer Cells Treated with Chemotherapeutic Agents. Cells.

[165] Hu X., Zhang H. (2019). Doxorubicin-Induced Cancer Cell Senescence Shows a Time Delay Effect and Is Inhibited by Epithelial-Mesenchymal Transition (EMT). Med. Sci. Monit..

[166] Hernandez-Segura A., Nehme J., Demaria M. (2018). Hallmarks of Cellular Senescence. Trends Cell. Biol..

[167] Dodig S., Čepelak I., Pavić I. (2019). Hallmarks of Senescence and Aging. Biochem. Med..

[168] Joyner D.E., Bastar J.D., Randall R.L. (2006). Doxorubicin Induces Cell Senescence Preferentially over Apoptosis in the FU-SY-1 Synovial Sarcoma Cell Line. J. Orthop. Res..

[169] Strzeszewska A., Alster O., Mosieniak G., Ciolko A., Sikora E. (2018). Insight into the Role of PIKK Family Members and NF-KB in DNAdamage-Induced Senescence and Senescence-Associated Secretory Phenotype of Colon Cancer Cells. Cell Death Dis..

[170] Roninson I.B. (2003). Tumor Cell Senescence in Cancer Treatment. Cancer Res..

[171] Yang L., Fang J., Chen J. (2017). Tumor Cell Senescence Response Produces Aggressive Variants. Cell Death Discov..

[172] Sliwinska M.A., Mosieniak G., Wolanin K., Babik A., Piwocka K., Magalska A., Szczepanowska J., Fronk J., Sikora E. (2009). Induction of Senescence with Doxorubicin Leads to Increased Genomic Instability of HCT116 Cells. Mech. Ageing Dev..

[173] Karabicici M., Alptekin S., Fırtına Karagonlar Z., Erdal E. (2021). Doxorubicin-Induced Senescence Promotes Stemness and Tumorigenicity in EpCAM-/CD133- Nonstem Cell Population in Hepatocellular Carcinoma Cell Line, HuH-7. Mol. Oncol..

[174] Yang M.-Y., Lin P.-M., Liu Y.-C., Hsiao H.-H., Yang W.-C., Hsu J.-F., Hsu C.-M., Lin S.-F. (2012). Induction of Cellular Senescence by Doxorubicin Is Associated with Upregulated MiR-375 and Induction of Autophagy in K562 Cells. PLoS ONE.

[175] Bientinesi E., Lulli M., Becatti M., Ristori S., Margheri F., Monti D. (2022). Doxorubicin-Induced Senescence in Normal Fibroblasts Promotes in Vitro Tumour Cell Growth and Invasiveness: The Role of Quercetin in Modulating These Processes. Mech. Ageing Dev..

[176] Marques L., Johnson A.A., Stolzing A. (2020). Doxorubicin Generates Senescent Microglia That Exhibit Altered Proteomes, Higher Levels of Cytokine Secretion, and a Decreased Ability to Internalize Amyloid β. Exp. Cell Res..

[177] Piegari E., De Angelis A., Cappetta D., Russo R., Esposito G., Costantino S., Graiani G., Frati C., Prezioso L., Berrino L. (2013). Doxorubicin Induces Senescence and Impairs Function of Human Cardiac Progenitor Cells. Basic Res. Cardiol..

[178] Spallarossa P., Altieri P., Aloi C., Garibaldi S., Barisione C., Ghigliotti G., Fugazza G., Barsotti A., Brunelli C. (2009). Doxorubicin Induces Senescence or Apoptosis in Rat Neonatal Cardiomyocytes by Regulating the Expression Levels of the Telomere Binding Factors 1 and 2. Am. J. Physiol. Heart Circ. Physiol..

[179] Mitry M.A., Laurent D., Keith B.L., Sira E., Eisenberg C.A., Eisenberg L.M., Joshi S., Gupte S., Edwards J.G. (2020). Accelerated Cardiomyocyte Senescence Contributes to Late-Onset Doxorubicin-Induced Cardiotoxicity. Am. J. Physiol. Cell Physiol..

[180] Bashiri Dezfouli A., Salar-Amoli J., Pourfathollah A.A., Yazdi M., Nikougoftar-Zarif M., Khosravi M., Hassan J. (2020). Doxorubicin-Induced Senescence through NF-ΚB Affected by the Age of Mouse Mesenchymal Stem Cells. J. Cell. Physiol..

[181] Sultana R., Di Domenico F., Tseng M., Cai J., Noel T., Chelvarajan R.L., Pierce W.D., Cini C., Bondada S., St Clair D.K. (2010). Doxorubicin-Induced Thymus Senescence. J. Proteome Res..

[182] Mathew R., Karantza-Wadsworth V., White E. (2007). Role of Autophagy in Cancer. Nat. Rev. Cancer.

[183] Zhao D., Yuan H., Yi F., Meng C., Zhu Q. (2014). Autophagy Prevents Doxorubicin-induced Apoptosis in Osteosarcoma. Mol. Med. Rep..

[184] Guo B., Tam A., Santi S.A., Parissenti A.M. (2016). Role of Autophagy and Lysosomal Drug Sequestration in Acquired Resistance to Doxorubicin in MCF-7 Cells. BMC Cancer.

[185] Sishi B.J.N., Loos B., van Rooyen J., Engelbrecht A.-M. (2013). Autophagy Upregulation Promotes Survival and Attenuates Doxorubicin-Induced Cardiotoxicity. Biochem. Pharmacol..

[186] Loh J.S., Rahim N.A., Tor Y.S., Foo J.B. (2022). Simultaneous Proteasome and Autophagy Inhibition Synergistically Enhances Cytotoxicity of Doxorubicin in Breast Cancer Cells. Cell Biochem. Funct..

[187] Aydinlik S., Erkisa M., Cevatemre B., Sarimahmut M., Dere E., Ari F., Ulukaya E. (2017). Enhanced Cytotoxic Activity of Doxorubicin through the Inhibition of Autophagy in Triple Negative Breast Cancer Cell Line. Biochim. Biophys. Acta Gen. Subj..

[188] Hu C., Gu F., Gong C., Xia Q., Gao Y., Gao S. (2022). Co-Delivery of the Autophagy Inhibitor Si-Beclin1 and the Doxorubicin Nano-Delivery System for Advanced Prostate Cancer Treatment. J. Biomater. Appl..

[189] Christidi E., Brunham L.R. (2021). Regulated Cell Death Pathways in Doxorubicin-Induced Cardiotoxicity. Cell Death Dis..

[190] Yang R., Li Y., Wang X., Yan J., Pan D., Xu Y., Wang L., Yang M. (2019). Doxorubicin Loaded Ferritin Nanoparticles for Ferroptosis Enhanced Targeted Killing of Cancer Cells. RSC Adv..

[191] Xue C.-C., Li M.-H., Zhao Y., Zhou J., Hu Y., Cai K.-Y., Zhao Y., Yu S.-H., Luo Z. (2020). Tumor Microenvironment-Activatable Fe-Doxorubicin Preloaded Amorphous CaCO3 Nanoformulation Triggers Ferroptosis in Target Tumor Cells. Sci. Adv..

[192] Yang Y., Zuo S., Li L., Kuang X., Li J., Sun B., Wang S., He Z., Sun J. (2021). Iron-Doxorubicin Prodrug Loaded Liposome Nanogenerator Programs Multimodal Ferroptosis for Efficient Cancer Therapy. Asian J. Pharm. Sci..

[193] Ji P., Wang X., Yin J., Yao Y., Du W. (2022). Amplification of Ferroptosis with a Liposomal Nanoreactor Cooperates with Low-Toxicity Doxorubicin Apoptosis for Enhanced Tumor Chemotherapy. Biomater. Sci..

[194] Bano I., Horky P., Abbas S.Q., Majid M., Bilal A.H.M., Ali F., Behl T., Shams ul Hassan S., Bungau S. (2022). Ferroptosis: A New Road towards Cancer Management. Molecules.

[195] Wang L., Qin X., Liang J., Ge P. (2021). Induction of Pyroptosis: A Promising Strategy for Cancer Treatment. Front. Oncol..

[196] Xia X., Wang X., Cheng Z., Qin W., Lei L., Jiang J., Hu J. (2019). The Role of Pyroptosis in Cancer: Pro-Cancer or pro-“Host”?. Cell Death Dis..

[197] Meng L., Lin H., Zhang J., Lin N., Sun Z., Gao F., Luo H., Ni T., Luo W., Chi J. (2019). Doxorubicin Induces Cardiomyocyte Pyroptosis via the TINCR-Mediated Posttranscriptional Stabilization of NLR Family Pyrin Domain Containing 3. J. Mol. Cell. Cardiol..

[198] Dessouki F.B.A., Kukreja R.C., Singla D.K. (2020). Stem Cell-Derived Exosomes Ameliorate Doxorubicin-Induced Muscle Toxicity through Counteracting Pyroptosis. Pharmaceuticals.

[199] Zhang Z., Zhang H., Li D., Zhou X., Qin Q., Zhang Q. (2021). Caspase-3-Mediated GSDME Induced Pyroptosis in Breast Cancer Cells through the ROS/JNK Signalling Pathway. J. Cell. Mol. Med..

[200] Yu P., Wang H.-Y., Tian M., Li A.-X., Chen X.-S., Wang X.-L., Zhang Y., Cheng Y. (2019). Eukaryotic Elongation Factor-2 Kinase Regulates the Cross-Talk between Autophagy and Pyroptosis in Doxorubicin-Treated Human Melanoma Cells in Vitro. Acta Pharmacol. Sin..

[201] Konstantinidou M., Zarganes-Tzitzikas T., Magiera-Mularz K., Holak T.A., Dömling A. (2018). Immune Checkpoint PD-1/PD-L1: Is There Life Beyond Antibodies?. Angew. Chem. Int. Ed. Engl..

[202] Terenzi A., Pirker C., Keppler B.K., Berger W. (2016). Anticancer Metal Drugs and Immunogenic Cell Death. J. Inorg. Biochem..

[203] Kythreotou A., Siddique A., Mauri F.A., Bower M., Pinato D.J. (2018). PD-L1. J. Clin. Pathol..

[204] Galluzzi L., Buqué A., Kepp O., Zitvogel L., Kroemer G. (2015). Immunological Effects of Conventional Chemotherapy and Targeted Anticancer Agents. Cancer Cell.

[205] Mattarollo S.R., Loi S., Duret H., Ma Y., Zitvogel L., Smyth M.J. (2011). Pivotal Role of Innate and Adaptive Immunity in Anthracycline Chemotherapy of Established Tumors. Cancer Res..

[206] Park J.Y., Jang M.J., Chung Y.H., Kim K.Y., Kim S.S., Lee W.B., You S., Choi Y.S., Hur D.Y., Kim D. (2009). Doxorubicin Enhances CD4(+) T-Cell Immune Responses by Inducing Expression of CD40 Ligand and 4-1BB. Int. Immunopharmacol..

[207] Zirakzadeh A.A., Kinn J., Krantz D., Rosenblatt R., Winerdal M.E., Hu J., Hartana C.A., Lundgren C., Bergman E.A., Johansson M. (2017). Doxorubicin Enhances the Capacity of B Cells to Activate T Cells in Urothelial Urinary Bladder Cancer. Clin. Immunol..

[208] Bedi D., Henderson H.J., Manne U., Samuel T. (2019). Camptothecin Induces PD-L1 and Immunomodulatory Cytokines in Colon Cancer Cells. Medicines.

[209] Gilad Y., Eliaz Y., Yu Y., Han S.J., O’Malley B.W., Lonard D.M. (2019). Drug-Induced PD-L1 Expression and Cell Stress Response in Breast Cancer Cells Can Be Balanced by Drug Combination. Sci. Rep..

[210] Voorwerk L., Slagter M., Horlings H.M., Sikorska K., van de Vijver K.K., de Maaker M., Nederlof I., Kluin R.J.C., Warren S., Ong S. (2019). Immune Induction Strategies in Metastatic Triple-Negative Breast Cancer to Enhance the Sensitivity to PD-1 Blockade: The TONIC Trial. Nat. Med..

[211] Wang J., Hu C., Wang J., Shen Y., Bao Q., He F., Wang H., Gong L., Liu Z., Hu F. (2019). Checkpoint Blockade in Combination With Doxorubicin Augments Tumor Cell Apoptosis in Osteosarcoma. J. Immunother..

[212] Iwai T., Sugimoto M., Wakita D., Yorozu K., Kurasawa M., Yamamoto K. (2018). Topoisomerase I Inhibitor, Irinotecan, Depletes Regulatory T Cells and up-Regulates MHC Class I and PD-L1 Expression, Resulting in a Supra-Additive Antitumor Effect When Combined with Anti-PD-L1 Antibodies. Oncotarget.

[213] Zhu X., Xu J., Cai H., Lang J. (2018). Carboplatin and Programmed Death-Ligand 1 Blockade Synergistically Produce a Similar Antitumor Effect to Carboplatin Alone in Murine ID8 Ovarian Cancer Model. J. Obstet. Gynaecol. Res..

[214] Wahba J., Natoli M., Whilding L.M., Parente-Pereira A.C., Jung Y., Zona S., Lam E.W.-F., Smith J.R., Maher J., Ghaem-Maghami S. (2018). Chemotherapy-Induced Apoptosis, Autophagy and Cell Cycle Arrest Are Key Drivers of Synergy in Chemo-Immunotherapy of Epithelial Ovarian Cancer. Cancer Immunol. Immunother..

[215] Fournel L., Wu Z., Stadler N., Damotte D., Lococo F., Boulle G., Ségal-Bendirdjian E., Bobbio A., Icard P., Trédaniel J. (2019). Cisplatin Increases PD-L1 Expression and Optimizes Immune Check-Point Blockade in Non-Small Cell Lung Cancer. Cancer Lett..

[216] Tran L., Allen C.T., Xiao R., Moore E., Davis R., Park S.-J., Spielbauer K., Van Waes C., Schmitt N.C. (2017). Cisplatin Alters Antitumor Immunity and Synergizes with PD-1/PD-L1 Inhibition in Head and Neck Squamous Cell Carcinoma. Cancer Immunol. Res..

[217] Ock C.-Y., Kim S., Keam B., Kim S., Ahn Y.-O., Chung E.-J., Kim J.-H., Kim T.M., Kwon S.K., Jeon Y.K. (2017). Changes in Programmed Death-Ligand 1 Expression during Cisplatin Treatment in Patients with Head and Neck Squamous Cell Carcinoma. Oncotarget.

[218] Tsai T.-F., Lin J.-F., Lin Y.-C., Chou K.-Y., Chen H.-E., Ho C.-Y., Chen P.-C., Hwang T.I.-S. (2019). Cisplatin Contributes to Programmed Death-Ligand 1 Expression in Bladder Cancer through ERK1/2-AP-1 Signaling Pathway. Biosci. Rep..

[219] Qin X., Liu C., Zhou Y., Wang G. (2010). Cisplatin Induces Programmed Death-1-Ligand 1(PD-L1) over-Expression in Hepatoma H22 Cells via Erk /MAPK Signaling Pathway. Cell. Mol. Biol..

[220] Wu X., Li Y., Liu X., Chen C., Harrington S.M., Cao S., Xie T., Pham T., Mansfield A.S., Yan Y. (2018). Targeting B7-H1 (PD-L1) Sensitizes Cancer Cells to Chemotherapy. Heliyon.

[221] Sato H., Niimi A., Yasuhara T., Permata T.B.M., Hagiwara Y., Isono M., Nuryadi E., Sekine R., Oike T., Kakoti S. (2017). DNA Double-Strand Break Repair Pathway Regulates PD-L1 Expression in Cancer Cells. Nat. Commun..

[222] Doi T., Ishikawa T., Okayama T., Oka K., Mizushima K., Yasuda T., Sakamoto N., Katada K., Kamada K., Uchiyama K. (2017). The JAK/STAT Pathway Is Involved in the Upregulation of PD-L1 Expression in Pancreatic Cancer Cell Lines. Oncol. Rep..

[223] Chen B., Hu J., Hu X., Chen H., Bao R., Zhou Y., Ye Y., Zhan M., Cai W., Li H. (2022). DENR Controls JAK2 Translation to Induce PD-L1 Expression for Tumor Immune Evasion. Nat. Commun..

[224] Zhao T., Li Y., Zhang J., Zhang B. (2020). PD-L1 Expression Increased by IFN-γ via JAK2-STAT1 Signaling and Predicts a Poor Survival in Colorectal Cancer. Oncol. Lett..

[225] Zhu J., Li Y., Lv X. (2022). IL4I1 Enhances PD-L1 Expression through JAK/STAT Signaling Pathway in Lung Adenocarcinoma. Immunogenetics.

[226] Black M., Barsoum I.B., Truesdell P., Cotechini T., Macdonald-Goodfellow S.K., Petroff M., Siemens D.R., Koti M., Craig A.W.B., Graham C.H. (2016). Activation of the PD-1/PD-L1 Immune Checkpoint Confers Tumor Cell Chemoresistance Associated with Increased Metastasis. Oncotarget.

[227] Seetharamu N., Preeshagul I.R., Sullivan K.M. (2017). New PD-L1 Inhibitors in Non-Small Cell Lung Cancer—Impact of Atezolizumab. Lung Cancer.

[228] Liu X., Guo C.-Y., Tou F.-F., Wen X.-M., Kuang Y.-K., Zhu Q., Hu H. (2020). Association of PD-L1 Expression Status with the Efficacy of PD-1/PD-L1 Inhibitors and Overall Survival in Solid Tumours: A Systematic Review and Meta-Analysis. Int. J. Cancer.

[229] Liu S., Chen S., Yuan W., Wang H., Chen K., Li D., Li D. (2017). PD-1/PD-L1 Interaction up-Regulates MDR1/P-Gp Expression in Breast Cancer Cells via PI3K/AKT and MAPK/ERK Pathways. Oncotarget.

[230] Emami F., Banstola A., Vatanara A., Lee S., Kim J.O., Jeong J.-H., Yook S. (2019). Doxorubicin and Anti-PD-L1 Antibody Conjugated Gold Nanoparticles for Colorectal Cancer Photochemotherapy. Mol. Pharm..

[231] Bailly C., Thuru X., Quesnel B. (2020). Combined Cytotoxic Chemotherapy and Immunotherapy of Cancer: Modern Times. NAR Cancer.

[232] Yang M., Liu P., Wang K., Glorieux C., Hu Y., Wen S., Jiang W., Huang P. (2017). Chemotherapy Induces Tumor Immune Evasion by Upregulation of Programmed Cell Death Ligand 1 Expression in Bone Marrow Stromal Cells. Mol. Oncol..

[233] Lammers T., Kiessling F., Hennink W.E., Storm G. (2012). Drug Targeting to Tumors: Principles, Pitfalls and (Pre-) Clinical Progress. J. Control. Release.

[234] Coimbra M., Crielaard B.J., Storm G., Schiffelers R.M. (2012). Critical Factors in the Development of Tumor-Targeted Anti-Inflammatory Nanomedicines. J. Control. Release.

[235] Akbarzadeh A., Rezaei-Sadabady R., Davaran S., Joo S.W., Zarghami N., Hanifehpour Y., Samiei M., Kouhi M., Nejati-Koshki K. (2013). Liposome: Classification, Preparation, and Applications. Nanoscale Res. Lett..

[236] Liu P., Chen G., Zhang J. (2022). A Review of Liposomes as a Drug Delivery System: Current Status of Approved Products, Regulatory Environments, and Future Perspectives. Molecules.

[237] Mishra P., Nayak B., Dey R.K. (2016). PEGylation in Anti-Cancer Therapy: An Overview. Asian J. Pharm. Sci..

[238] Hussain Z., Khan S., Imran M., Sohail M., Shah S.W.A., de Matas M. (2019). PEGylation: A Promising Strategy to Overcome Challenges to Cancer-Targeted Nanomedicines: A Review of Challenges to Clinical Transition and Promising Resolution. Drug Deliv. Transl. Res..

[239] Eavarone D.A., Yu X., Bellamkonda R.V. (2000). Targeted Drug Delivery to C6 Glioma by Transferrin-Coupled Liposomes. J. Biomed. Mater. Res..

[240] Paszko E., Senge M.O. (2012). Immunoliposomes. Curr. Med. Chem..

[241] Sapra P., Moase E.H., Ma J., Allen T.M. (2004). Improved Therapeutic Responses in a Xenograft Model of Human B Lymphoma (Namalwa) for Liposomal Vincristine versus Liposomal Doxorubicin Targeted via Anti-CD19 IgG2a or Fab’ Fragments. Clin. Cancer Res..

[242] Sapra P., Allen T.M. (2002). Internalizing Antibodies Are Necessary for Improved Therapeutic Efficacy of Antibody-Targeted Liposomal Drugs. Cancer Res..

[243] Cheng W.W.K., Allen T.M. (2008). Targeted Delivery of Anti-CD19 Liposomal Doxorubicin in B-Cell Lymphoma: A Comparison of Whole Monoclonal Antibody, Fab’ Fragments and Single Chain Fv. J. Control. Release.

[244] Tedder T.F., Poe J.C., Haas K.M. (2005). CD22: A Multifunctional Receptor That Regulates B Lymphocyte Survival and Signal Transduction. Adv. Immunol..

[245] Tuscano J.M., Martin S.M., Ma Y., Zamboni W., O’Donnell R.T. (2010). Efficacy, Biodistribution, and Pharmacokinetics of CD22-Targeted Pegylated Liposomal Doxorubicin in a B-Cell Non-Hodgkin’s Lymphoma Xenograft Mouse Model. Clin. Cancer Res..

[246] O’Donnell R.T., Martin S.M., Ma Y., Zamboni W.C., Tuscano J.M. (2010). Development and Characterization of CD22-Targeted Pegylated-Liposomal Doxorubicin (IL-PLD). Investig. New Drugs.

[247] Batist G., Barton J., Chaikin P., Swenson C., Welles L. (2002). Myocet (Liposome-Encapsulated Doxorubicin Citrate): A New Approach in Breast Cancer Therapy. Expert. Opin. Pharmacother..

[248] Swenson C.E., Bolcsak L.E., Batist G., Guthrie T.H., Tkaczuk K.H., Boxenbaum H., Welles L., Chow S.-C., Bhamra R., Chaikin P. (2003). Pharmacokinetics of Doxorubicin Administered i.v. as Myocet (TLC D-99; Liposome-Encapsulated Doxorubicin Citrate) Compared with Conventional Doxorubicin When given in Combination with Cyclophosphamide in Patients with Metastatic Breast Cancer. Anticancer Drugs.

[249] Mross K., Niemann B., Massing U., Drevs J., Unger C., Bhamra R., Swenson C.E. (2004). Pharmacokinetics of Liposomal Doxorubicin (TLC-D99; Myocet) in Patients with Solid Tumors: An Open-Label, Single-Dose Study. Cancer Chemother. Pharmacol..

[250] Dell’Olio M., Scalzulli R.P., Sanpaolo G., Nobile M., Mantuano F.S., La Sala A., D’Arena G., Miraglia E., Lucania A., Mastrullo L. (2011). Non-Pegylated Liposomal Doxorubicin (Myocet^®^) in Patients with Poor-Risk Aggressive B-Cell Non-Hodgkin Lymphoma. Leuk. Lymphoma.

[251] Viale M., Bertone V., Maric I., Cilli M., Emionite L., Bocchini V., Ponzoni M., Fontana V., De Luca F., Rocco M. (2021). Enhanced Therapeutic Index of Liposomal Doxorubicin Myocet Locally Delivered by Fibrin Gels in Immunodeficient Mice Bearing Human Neuroblastoma. Pharmacol. Res..

[252] Burade V., Bhowmick S., Maiti K., Zalawadia R., Ruan H., Thennati R. (2017). Lipodox^®^ (Generic Doxorubicin Hydrochloride Liposome Injection): In Vivo Efficacy and Bioequivalence versus Caelyx^®^ (Doxorubicin Hydrochloride Liposome Injection) in Human Mammary Carcinoma (MX-1) Xenograft and Syngeneic Fibrosarcoma (WEHI 164) Mouse Models. BMC Cancer.

[253] Smith J.A., Costales A.B., Jaffari M., Urbauer D.L., Frumovitz M., Kutac C.K., Tran H., Coleman R.L. (2016). Is It Equivalent? Evaluation of the Clinical Activity of Single Agent Lipodox^®^ Compared to Single Agent Doxil^®^ in Ovarian Cancer Treatment. J. Oncol. Pharm. Pract..

[254] Smith J.A., Mathew L., Burney M., Nyshadham P., Coleman R.L. (2016). Equivalency Challenge: Evaluation of Lipodox^®^ as the Generic Equivalent for Doxil^®^ in a Human Ovarian Cancer Orthotropic Mouse Model. Gynecol. Oncol..

[255] Sehouli J., Camara O., Schmidt M., Mahner S., Seipelt G., Otremba B., Schmalfeldt B., Tesch H., Lorenz-Schlüter C., Oskay-Ozcelik G. (2009). Pegylated Liposomal Doxorubicin (CAELYX) in Patients with Advanced Ovarian Cancer: Results of a German Multicenter Observational Study. Cancer Chemother. Pharmacol..

[256] Dellapasqua S., Trillo Aliaga P., Munzone E., Bagnardi V., Pagan E., Montagna E., Cancello G., Ghisini R., Sangalli C., Negri M. (2021). Pegylated Liposomal Doxorubicin (Caelyx^®^) as Adjuvant Treatment in Early-Stage Luminal B-like Breast Cancer: A Feasibility Phase II Trial. Curr. Oncol..

[257] Dou Y., Hynynen K., Allen C. (2017). To Heat or Not to Heat: Challenges with Clinical Translation of Thermosensitive Liposomes. J. Control. Release.

[258] Besse H.C., Barten-van Rijbroek A.D., van der Wurff-Jacobs K.M.G., Bos C., Moonen C.T.W., Deckers R. (2019). Tumor Drug Distribution after Local Drug Delivery by Hyperthermia, In Vivo. Cancers.

[259] Rajappa S., Joshi A., Doval D.C., Batra U., Rajendranath R., Deo A., Biswas G., Bajpai P., Tilak T.V.S., Kane S. (2018). Novel Formulations of Docetaxel, Paclitaxel and Doxorubicin in the Management of Metastatic Breast Cancer. Oncol. Lett..

[260] Li J., Zhang B., Yue C., Wu J., Zhao L., Sun D., Wang R. (2018). Strategies to Release Doxorubicin from Doxorubicin Delivery Vehicles. J. Drug Target..

[261] Birngruber T., Raml R., Gladdines W., Gatschelhofer C., Gander E., Ghosh A., Kroath T., Gaillard P.J., Pieber T.R., Sinner F. (2014). Enhanced Doxorubicin Delivery to the Brain Administered through Glutathione PEGylated Liposomal Doxorubicin (2B3-101) as Compared with Generic Caelyx,(^®^)/Doxil(^®^)--a Cerebral Open Flow Microperfusion Pilot Study. J. Pharm. Sci..

[262] Gaillard P.J., Appeldoorn C.C.M., Dorland R., van Kregten J., Manca F., Vugts D.J., Windhorst B., van Dongen G.A.M.S., de Vries H.E., Maussang D. (2014). Pharmacokinetics, Brain Delivery, and Efficacy in Brain Tumor-Bearing Mice of Glutathione Pegylated Liposomal Doxorubicin (2B3-101). PLoS ONE.

[263] Mehrabian A., Vakili-Ghartavol R., Mashreghi M., Shokooh Saremi S., Badiee A., Arabi L., Alavizadeh S.H., Moosavian S.A., Jaafari M.R. (2022). Preparation, Characterization, and Biodistribution of Glutathione PEGylated Nanoliposomal Doxorubicin for Brain Drug Delivery with a Post-Insertion Approach. Iran. J. Basic Med. Sci..

[264] Fraguas-Sánchez A.I., Lozza I., Torres-Suárez A.I. (2022). Actively Targeted Nanomedicines in Breast Cancer: From Pre-Clinal Investigation to Clinic. Cancers.

[265] Makwana V., Karanjia J., Haselhorst T., Anoopkumar-Dukie S., Rudrawar S. (2021). Liposomal Doxorubicin as Targeted Delivery Platform: Current Trends in Surface Functionalization. Int. J. Pharm..

[266] Luo R., Li Y., He M., Zhang H., Yuan H., Johnson M., Palmisano M., Zhou S., Sun D. (2017). Distinct Biodistribution of Doxorubicin and the Altered Dispositions Mediated by Different Liposomal Formulations. Int. J. Pharm..

[267] Mastria E.M., Cai L.Y., Kan M.J., Li X., Schaal J.L., Fiering S., Gunn M.D., Dewhirst M.W., Nair S.K., Chilkoti A. (2018). Nanoparticle Formulation Improves Doxorubicin Efficacy by Enhancing Host Antitumor Immunity. J. Control. Release.

[268] Hannesdóttir L., Tymoszuk P., Parajuli N., Wasmer M.-H., Philipp S., Daschil N., Datta S., Koller J.-B., Tripp C.H., Stoitzner P. (2013). Lapatinib and Doxorubicin Enhance the Stat1-Dependent Antitumor Immune Response. Eur. J. Immunol..

[269] Eggleton P., Bremer E., Dudek E., Michalak M. (2016). Calreticulin, a Therapeutic Target?. Expert Opin. Ther. Targets.

[270] Kawano M., Tanaka K., Itonaga I., Iwasaki T., Miyazaki M., Ikeda S., Tsumura H. (2016). Dendritic Cells Combined with Doxorubicin Induces Immunogenic Cell Death and Exhibits Antitumor Effects for Osteosarcoma. Oncol. Lett..

[271] Bell C.W., Jiang W., Reich C.F., Pisetsky D.S. (2006). The Extracellular Release of HMGB1 during Apoptotic Cell Death. Am. J. Physiol. Cell Physiol..

[272] Schiller M., Heyder P., Ziegler S., Niessen A., Claßen L., Lauffer A., Lorenz H.-M. (2013). During Apoptosis HMGB1 Is Translocated into Apoptotic Cell-Derived Membranous Vesicles. Autoimmunity.

[273] Lee J.-J., Park I.H., Rhee W.J., Kim H.S., Shin J.-S. (2019). HMGB1 Modulates the Balance between Senescence and Apoptosis in Response to Genotoxic Stress. FASEB J..

[274] Yang S., Shim M.K., Kim W.J., Choi J., Nam G.-H., Kim J., Kim J., Moon Y., Kim H.Y., Park J. (2021). Cancer-Activated Doxorubicin Prodrug Nanoparticles Induce Preferential Immune Response with Minimal Doxorubicin-Related Toxicity. Biomaterials.

[275] Merino M., Lozano T., Casares N., Lana H., Troconiz I.F., Ten Hagen T.L.M., Kochan G., Berraondo P., Zalba S., Garrido M.J. (2021). Dual Activity of PD-L1 Targeted Doxorubicin Immunoliposomes Promoted an Enhanced Efficacy of the Antitumor Immune Response in Melanoma Murine Model. J. Nanobiotechnol..

[276] Wang W., Zhang L., Sun Z. (2022). Eliciting Pyroptosis to Fuel Cancer Immunotherapy: Mechanisms and Strategies. Cancer Biol. Med..

[277] Zhu L., Lin M. (2021). The Synthesis of Nano-Doxorubicin and Its Anticancer Effect. Anti-Cancer Agents Med. Chem..

[278] Ibrahim M., Abuwatfa W.H., Awad N.S., Sabouni R., Husseini G.A. (2022). Encapsulation, Release, and Cytotoxicity of Doxorubicin Loaded in Liposomes, Micelles, and Metal-Organic Frameworks: A Review. Pharmaceutics.

